# Demographic and conservation genomic assessment of the threatened marbled teal (*Marmaronetta angustirostris*)

**DOI:** 10.1111/eva.13639

**Published:** 2024-05-08

**Authors:** Joaquín Ortego, Violeta Muñoz‐Fuentes, Raquel López‐Luque, Alex D. Ball, Muhammad Ghazali, Salwan Ali Abed, Mudhafar A. Salim, Andy J. Green

**Affiliations:** ^1^ Department of Ecology and Evolution Estación Biológica de Doñana, EBD‐CSIC Seville Spain; ^2^ European Molecular Biology Laboratory‐European Bioinformatics Institute Hinxton UK; ^3^ Department of Conservation Biology and Global Change Estación Biológica de Doñana, EBD‐CSIC Seville Spain; ^4^ RZSS WildGenes Laboratory Royal Zoological Society of Scotland Edinburgh Scotland UK; ^5^ Department of Environment, College of Science University of Al‐Qadisiyah Al Diwaniyah Iraq; ^6^ Iraqi Organization for Conservation of Nature (IOCN) Baghdad Iraq; ^7^ Present address: UN Environment World Conservation Monitoring Centre (UNEP‐WCMC) Cambridge UK

**Keywords:** conservation genetics, conservation‐breeding programs, demographic bottleneck, genetic diversity, introgression, marbled teal, *Marmaronetta angustirostris*

## Abstract

Genetic assessment of species that have experienced dramatic population declines provides critical information that is instrumental for the design of conservation recovery programs. Here, we use different sources of molecular data (mtDNA and ddRAD‐seq) to evaluate the genetic status of wild and captive populations of marbled teal (*Marmaronetta angustirostris*), a duck species classified as critically endangered in Spain and near threatened at a global scale. First, we determined the evolutionary and demographic trajectories of the wild population from Spain and the currently much larger population from Iraq, which is also the documented source of European zoo stocks. Second, we evaluated the suitability of the different captive populations for ongoing restocking programs in Spain and assessed their potential impact on the genetic composition of wild populations. Populations from Spain and Iraq were assigned to distinct genetic clusters, albeit with an overall low level of genetic differentiation, in line with their recent divergence (<8000 years ago) and lack of phylogeographic structure in the species. Demogenomic inferences revealed that the two populations have experienced parallel demographic trajectories, with a marked bottleneck during the last glacial period followed by a sudden demographic expansion and stability since the onset of the Holocene. The wild population from Spain presented high levels of inbreeding, but we found no evidence of recent genetic bottlenecks compatible with the human‐driven decline of the species during the past century. The captive populations from the two Spanish centers involved in restocking programs showed genetic introgression from European zoos; however, we found limited evidence of introgression from the zoo genetic stock into the wild population from Spain, suggesting captive‐bred birds have limited breeding success in the wild. Our study illustrates how ex situ conservation programs should consider the genetic distinctiveness of populations when establishing breeding stocks and highlights the importance of genetically assessing captive populations prior to reinforcement actions.

## 1 INTRODUCTION

Anthropogenic activities have brought many species to the verge of extinction, which has led to the progressive implementation of protective legislation and management actions aimed at reversing population declines (Butchart et al., 2010). Determining the levels of genetic diversity of threatened species and understanding how such diversity is distributed across space and time is a cornerstone of conservation biology (Frankham, 1995, 2003). Such information is particularly relevant for vertebrates, which often sustain small effective population sizes compared to other organisms, are prone to genetic fragmentation, and experience the negative consequences of loss of genetic diversity over short spatiotemporal scales (Li et al., 2016; e.g., Casas‐Marce et al., 2013; Martínez‐Cruz et al., 2007; Muñoz‐Fuentes et al., 2005; Rubidge et al., 2012). Genetic monitoring of natural populations (Schwartz et al., 2007; e.g., Ortego et al., 2007, 2011; Thomas et al., 2022) and/or genomic‐based demographic reconstructions of changes in effective population size (*N*_e_) through time (e.g., Abascal et al., 2016; Stoffel et al., 2018) is key to determining whether documented declines in census population sizes (*N*_C_) have actually translated into the genetic erosion of contemporary populations. This has direct implications for understanding the future capacity of species to respond to selection pressures and adapt to climate warming and other environmental change conditions (Frankham, 1995; e.g., Stoffel et al., 2018).

Baseline information on species of conservation concern includes the genetic delineation of intraspecific units, a fundamental step for identifying evolutionary significant units (ESUs) and management units (MUs) for conservation aimed at preserving the ecological and evolutionary distinctiveness of populations (Fraser & Bernatchez, 2001; Moritz, 2002; e.g., Muñoz‐Fuentes et al., 2009; Yannic et al., 2016). This information is crucial for establishing captive stocks as sources for future reintroductions and reinforcement actions in ex situ conservation programs, which should consider the potential ecological and evolutionary idiosyncrasies of local populations (i.e., to avoid the disruption of local adaptations) and balance the deleterious consequences of both inbreeding and outbreeding depression on population viability (Witzenberger & Hochkirch, 2011; e.g., Kleinman‐Ruiz et al., 2019; Marshall & Spalton, 2000). However, although genetic assessments of endangered species is key for designing both in situ and ex situ conservation programs, such information is often limited or virtually absent, even for some of the best‐known threatened species (Stoffel et al., 2018; Yannic et al., 2014).

The marbled teal *Marmaronetta angustirostris* is a small duck with a range extending from north‐west Africa to western China, although the largest populations are centered around south‐west Asia and the western and eastern Mediterranean (Porter & Aspinall, 2010; Salvador et al., 2023; Wetlands International, 2023). Owing to its extensive range and nomadic movements, there has been much uncertainty about the overall size of biogeographical populations and the extent of population declines (Green, 2005). In recent decades, the species was considered as globally threatened with extinction, although the latest assessments using IUCN criteria have assigned it as near threatened at a global scale, and vulnerable in Europe (BirdLife International, 2021, 2022). Historical information suggests drastic population declines in many areas (Green, 1993), including Spain, which holds the majority of the European population, and where it is classified as Critically Endangered (Aranda et al., 1995; Giménez et al., 2021). Major declines in the marbled teal population in Spain had already been witnessed by 1950, and then became more acute by 1980 (Aranda et al., 1995; Green, 1993; Valverde, 1964). These declines were attributed to a combination of hunting pressure and habitat loss, especially drainage of seasonal wetlands. Wetland destruction and fragmentation in Spain and the rest of the Mediterranean region was already extensive by 1900, although it accelerated after 1950 (Green et al., 2002; Perennou et al., 2012; see, also, Fluet‐Chouinard et al., 2023). In contrast, the immense marshes of Iraq and Iran, associated with the deltas of the Tigris and Euphrates rivers, were largely intact until 1970, and still provide a much larger area of suitable habitat for marbled teals than is remaining in the western Mediterranean (Abed et al., 2014; BirdLife International, 2022; Green, 1993). Other factors such as lead poisoning are also likely to have contributed to population declines of the species (Svanberg et al., 2006).

Growing concern about population declines of marbled teal led to a series of international conservation plans, and the adoption of management measures, notably in Spain (Green, 1993; Green et al., 2004, 2005; Iñigo et al., 2008). Among others, conservation actions included the release of captive‐bred birds, which began in the 1990s and intensified since 2020, although records of how many birds were released, and their origins, are incomplete (Aranda et al., 1995; Fundación Biodiversidad, 2022; Pérez‐García et al., in press). The captive populations from the two Spanish centers currently involved in breeding programs for reinforcement—Cañada de los Pájaros (Andalusia Region) and Granja de El Saler (Valencia Region)—were putatively founded with native birds, but lack of long‐term monitoring of pairings or use of studbooks makes it difficult to trace their precise origin. European zoo populations were established with birds originating from the Wildfowl & Wetlands Trust (WWT) centers in the United Kingdom, which were founded in 1948 with only nine birds from Basra wetlands in Iraq (Hawkes, 1970; Jones, 1951). Different European zoos have also provided captive‐bred birds for release into wetlands in the Balearic Islands since 2015 (Fundación Biodiversidad, 2022). The availability of cheap birds on the European avicultural market might even have allowed private individuals to breed or release their own birds in Spain (Aranda et al., 1995). However, despite the disparate origins of birds used in reintroduction and reinforcement actions, we know virtually nothing about the genetic distinctiveness of their wild source populations. This is particularly important considering that populations from Spain and Iraq are assumed to belong to separate biogeographical (i.e., flyway) populations in the West Mediterranean and south‐west Asia, respectively (Salvador et al., 2023; Scott & Rose, 1996; Wetlands International, 2023).

Although the marbled teal is a species of conservation concern, no previous study has analyzed the genetic composition of wild and captive populations, determined their levels of genetic diversity, or evaluated the demographic trajectories (i.e., bottlenecks vs. stability) experienced by wild populations from different regions with contrasting census sizes and population trends. Neither has there been an assessment of whether the massive release of birds in Spain has resulted in genetic introgression of alien genotypes, with potential impacts on the genetic integrity and viability of native populations (BirdLife International, 2021; Fundación Biodiversidad, 2022). With increasing need for a One Plan approach to conservation planning and management (Byers et al., 2013), it is timely to join up the in situ and ex situ actions for the marbled teal.

Here, we apply complementary sources of molecular data—mtDNA sequences and genome‐wide nuclear markers—to determine the evolutionary and demographic trajectories of wild populations of marbled teal and evaluate the suitability of the different captive stocks for ongoing reinforcement programs. In a first step, we (i) compared the levels of genetic diversity and the demographic trajectories of wild populations from Spain and Iraq. Given that populations from south‐west Asia sustain much larger census sizes and have likely experienced less dramatic population declines in recent times than populations from the Western Mediterranean (BirdLife International, 2021), we hypothesize that the latter present lower levels of genetic diversity, increased inbreeding, and genomic signatures of recent population bottlenecks. In the second step, (ii) we evaluated the potential value and suitability of European captive populations for restocking programs ongoing in Spain. To this end, we determined the genetic composition of European captive populations and quantified the extent of divergence—or its lack thereof—between their respective source populations in Spain and Iraq. Our analyses revealed that captive breeding stocks and their respective wild source populations are assigned to different genetic clusters, supporting previous assumptions that populations from Spain and Iraq belong to distinctive biogeographical units. Given this, (iii) we assessed whether wild populations from Spain and captive populations from the two Spanish centers currently involved in reinforcement programs have experienced introgression from the genetic stock of European zoos established with birds from Iraq.

## 2 MATERIALS AND METHODS

### 2.1 Samples

#### 2.1.1 Samples from wild populations

We obtained tissue samples from fresh and museum specimens of wild marbled teal covering most of the historical and contemporary distribution range of the species (BirdLife International, 2021). Samples successfully amplified and sequenced (see Section 2.2) included specimens from Spain, Morocco, Tunisia, Algeria, Cape Verde, Chad, Israel, Iraq, and Iran (Table 1). Tissue samples from museum skins consisted of feathers or footpads, depending on the preference of museum curators, and included several specimens from Spain (*n* = 21) that predate (1967–1978) the massive release of captive‐bred birds in the country since 1990 (Aranda et al., 1995; Fundación Biodiversidad, 2022; Pérez‐García et al., in press). Fresh samples of contemporary birds (1991–2020) consisted of blood or muscle of wild specimens from Spain and Iraq. Samples from Spain were obtained from the Guadalquivir Marshes in south‐west Spain (in and around Doñana protected area) and multiple localities from Valencia region in south‐east Spain, mainly from El Hondo wetland complex in Alicante province. These samples included muscle from individuals found dead in the field, or which died after entering recovery centers, usually due to lead poisoning or disease outbreaks such as botulism. Blood or feather samples were also obtained from individuals captured during ongoing efforts to mark wild birds to study their movements. All these birds were considered “wild” because they were unringed when captured, but we cannot rule out the possibility that some of them were descendants of the many captive‐bred birds released in Spain over the past three decades (Fundación Biodiversidad, 2022; Pérez‐García et al., in press). Wild specimens from Iraq had been captured by local hunters and were on sale in a market. After purchasing the birds and taking a blood sample, they were released in Hawr Al‐Dalmaj wetlands, Iraq. As populations from south‐west Asia currently sustain much larger census sizes than those from the Western Mediterranean and have likely experienced less severe demographic declines in recent times (BirdLife International, 2021; Salim et al., 2009), samples from Iraq were used as a reference for genomic‐based demographic reconstructions (see Section 2.6). Iraq was also the reported source of birds captured in 1948 to establish a captive population at the Wildfowl & Wetlands Trust (WWT) Slimbridge (UK) from which, in turn, zoo populations in neighboring European countries were founded (Hawkes, 1970; Jones, 1951; see Section 2.1.2). Until needed for DNA extractions, feathers were stored in paper envelopes at room temperature, muscle samples were preserved in 96% ethanol at −20°C, and blood samples were preserved either on Whatman FTA Cards (Whatman Bioscience, Florham Park, NJ, USA) at room temperature or in 96% ethanol at −20°C.

**TABLE 1:** Captive and wild populations of marbled teal (*M. angustirostris*) analyzed in this study, including sample sizes (*n*) and genetic diversity statistics for both mtDNA (*H*, Hd, and *π*_;_ *n* ≥ 4) and ddRAD‐seq (*H*_O_, *H*_E_, *π*, and *F*_IS;_ *n* ≥ 4) datasets.

Population	Country^a^	Status	Year	Code	mtDNA dataset				ddRAD‐seq dataset				
					*n*	*H*	Hd	*π*	*n* ^b^	*H*_O_	*H*_E_	*π*	*F*_IS_
Wildfowl & Wetlands Trust Centers	UK	Captive	1948–2019	cWWTC	10	2	0.200	0.001	10 (5)	0.156	0.167	0.190	0.070
Tierpark Berlin	DE	Captive	2021	cTIER	8	1	0.000	0.000	8 (5)	0.155	0.155	0.177	0.046
Kölner Zoo	DE	Captive	2021	cKOLN	10	1	0.000	0.000	1 (1)	—	—	—	—
Opel Zoo	DE	Captive	2021	cOPEL	7	1	0.000	0.000	1 (1)	—	—	—	—
Gaia Zoo	NL	Captive	2019–2020	cGAIA	3	—	—	—	—	—	—	—	—
Parc Animalier de Sainte‐Croix	FR	Captive	2021	cCROI	8	1	0.000	0.000	8 (7)	0.112	0.129	0.140	0.061
Parc Animalier de Branféré	FR	Captive	2021	cBRAN	10	1	0.000	0.000	—	—	—	—	—
Walter Zoo	CH	Captive	2021	cWALT	4	1	0.000	0.000	4 (4)	0.150	0.145	0.174	0.047
Zoobotánico Jerez	ES	Captive	2018–2019	cJERE	12	1	0.000	0.000	12 (2)	—	—	—	—
Cañada de los Pájaros^c^	ES	Captive	2018–2021	cPAJA	20	2	0.526	0.006	14 (8)	0.171	0.175	0.188	0.047
La Granja de El Saler^c^	ES	Captive	2017–2018	cSALE	23	3	0.617	0.006	25 (6)	0.172	0.186	0.205	0.073
Guadalquivir Marshes^d^	ES	Wild	2005–2020	wGUAD	5	3	0.700	0.008	5 (4)	0.147	0.172	0.202	0.108
Guadalquivir Marshes^e^	ES	Wild	1967–1978	wGUAD	17	3	0.618	0.007	—	—	—	—	—
Valencia^d^	ES	Wild	1991–2020	wVALE	32	5	0.694	0.009	20 (20)	0.123	0.209	0.216	0.290
Castilla‐La Mancha^e^	ES	Wild	1970	wCAST	4	4	1.000	0.016	—	—	—	—	—
Morocco	MA	Wild	1926–1957	wMORO	3	—	—	—	—	—	—	—	—
Tunisia	TN	Wild	–	wTUNI	1	—	—	—	—	—	—	—	—
Algeria	DZ	Wild	1904	wALGE	1	—	—	—	—	—	—	—	—
Cape Verde	CV	Wild	1924	wCAPE	1	—	—	—	—	—	—	—	—
Chad	TD	Wild	1954	wCHAD	1	—	—	—	—	—	—	—	—
Israel	IL	Wild	1943–2009	wISRA	7	5	0.905	0.016	—	—	—	—	—
Iraq	IQ	Wild	2018	wIRAQ	12	4	0.652	0.010	12 (12)	0.206	0.215	0.226	0.064
Iran	IR	Wild	1937–1942	wIRAN	2	—	—	—	—	—	—	—	—

*Note*: Genetic diversity statistics for ddRAD‐seq data were calculated only considering unrelated individuals (*φ*_*ij*_ ≤0; see Figure 3) and variant (polymorphic) positions; statistics based on all positions (polymorphic and nonpolymorphic) are presented in Table S3.

Abbreviations: *F*_IS_, Wright's inbreeding coefficient; *H*, number of haplotypes; Hd, haplotype diversity; *H*_E_ expected heterozygosity; *H*_O_, observed heterozygosity; *π*, nucleotide diversity.

^a^ ISO country codes.

^b^ Figures in parentheses indicate sample sizes only considering unrelated individuals (*φ*_*ij*_ ≤ 0; see Figure 3).

^c^ Captive populations from Spanish centers currently involved in ex situ breeding programs for reinforcement.

^d^ Contemporary wild samples from Spain.

^e^ Historical wild samples from Spain predating the massive release of captive‐bred birds under reinforcement programs.

#### 2.1.2 Samples from captive populations

In order to characterize the genetic stocks of captive populations that are used or could be used for restocking programs, and evaluate the potential hybridization/introgression of native populations of marbled teal from Spain with birds originating from European zoos, we obtained samples from eleven captive populations of the species. These included samples from two WWT centers (Slimbridge and London) in the United Kingdom (https://www.wwt.org.uk) and eight zoos from Germany, the Netherlands, France, Switzerland and Spain. From here on, we refer to these captive populations as “European zoos”. As the two WWT centers are managed jointly and regularly exchange birds, they were treated as a single captive population in our study (Table 1). Furthermore, we obtained samples from the two captive breeding populations in Spain (Cañada de los Pájaros and La Granja de El Saler) currently involved in reinforcement programs within Spain (Table 1). These captive populations were putatively founded with wild individuals from Spain (Fundación Biodiversidad, 2022). Except for a museum specimen corresponding to one of the original birds captured in Iraq and used to found the WWT population in 1948, all samples from captive birds were fresh tissues (blood or feathers) from contemporary individuals (2017–2021) (Table 1). Samples were preserved as detailed in Section 2.1.1.

### 2.2 Molecular data

We used Qiagen DNeasy kits (Qiagen, Hilden, Germany) to extract and purify genomic DNA from each sample following the manufacturer's protocol for animal tissues. In the case of feathers, 20 μL of dithiothreitol (DTT) 1 M was added to the digestion buffer to achieve complete digestion. Most contemporary and all museum specimens were sequenced for a fragment of the control region of the mitochondrial DNA (Table 1; Section 2.2.1). All museum samples were processed in the low‐copy‐DNA laboratory from Estación Biológica de Doñana (EBD‐CSIC), where extant DNA and/or PCR products are never introduced, laboratory equipment and reagents are regularly sterilized, and dedicated protective clothing is worn. Given the low quality of DNA isolates from museum specimens, only DNA extracts from fresh tissues of contemporary individuals could be processed into genomic libraries to obtain genome‐wide data using the double‐digestion restriction‐site associated DNA sequencing (ddRAD‐seq) procedure (adapted from Peterson et al. (2012)) (Table 1; Section 2.2.2).

#### 2.2.1 mtDNA data

We designed primers L135.MaAn (5′‐GTCCCAGTAATACCCATTACCAG‐3′) and H614.MaAn (5′‐TGGAGGATGCCGCGATTACG‐3′) to amplify a 440 bp fragment of the mtDNA control region. For museum specimens, we designed primer H346.MaAn (5′‐TAGTGGTTGTCGGGGTATGTCC‐3′) which, combined with primer L135.MaAn, amplified a 169 bp fragment containing most polymorphic sites present in the 440 bp sequences obtained for contemporary samples. Method S1 provides all details on mtDNA amplification and sequencing. We trimmed, aligned and edited the sequences using geneious v. 2022.2.2 (http://www.geneious.com/). All downstream analyses are based on the short fragment available for both museum and contemporary samples.

#### 2.2.2 ddRAD‐seq data

DNA samples from contemporary samples were processed following the double‐digestion restriction‐site associated DNA sequencing procedure (ddRAD‐seq) adapted from Peterson et al. (2012), as detailed in Methods S2. We used stacks v. 2.62 to assemble our sequences into de novo loci and call genotypes (Rochette et al., 2019), a pipeline that produces datasets consistent with those obtained using reference genome‐based mapping approaches (Shafer et al., 2017; see also Fitz‐Gibbon et al., 2017). Unless otherwise indicated, for all downstream analyses, we exported only one random SNP per RAD locus (option *write‐random‐snp*) and retained loci that were represented in at least 75% of individuals (*R* = 0.75) and with a minimum minor allele frequency (MAF) ≥ 0.01 (*min_maf* = 0.01). For more details on assembling and filtering of sequence data, see Methods S3. Finally, we used the option *relatedness2* in vcftools (Danecek et al., 2011) to calculate the relatedness (kinship coefficient, *φ*_*i,j*_; Manichaikul et al., 2010) among all pairs of genotyped individuals and identify those with a kinship relationship (*φ*_*i,j*_ > 0).

### 2.3 Analyses of the mtDNA data

#### 2.3.1 Genetic diversity

We used dnasp v. 6.12.03 (Rozas et al., 2017) to determine the number of haplotypes (*H*), calculate haplotype diversity (Hd) and nucleotide diversity (π) for each population, and compare levels of genetic diversity between historical (1967–1978) and contemporary (1991–2020) samples from Spain. Statistical differences in genetic diversity between populations were tested by Welch's *t*‐tests in graphpad prism v. 8.8 (GraphPad Software Inc.). We also used dnasp to perform Tajima's *D* (Tajima, 1989) tests and examine deviations from neutral equilibrium that might be indicative of population expansions after a recent bottleneck (Tajima's *D* < 0) or sudden population contractions (Tajima's *D* > 0).

#### 2.3.2 Genetic differentiation and structure

We estimated a haplotype network using the TCS algorithm (Clement et al., 2000) as implemented in popart v. 1.7 (Leigh & Bryant, 2015). We used arlequin v. 3.5 (Excoffier & Lischer, 2010) to calculate *Φ*_ST_ values (an analogue of *F*_ST_ for haplotype sequence similarity) between each pair of wild populations (*n* ≥ 4; Table 1) and contemporary and historical samples from Spain, determining statistical significance with Fisher's exact tests after 10,000 permutations. Finally, we ran an analysis of molecular variance (AMOVA) to analyze the distribution of genetic variation among the three population groups delimited by Scott and Rose (1996), namely (i) west Mediterranean and west Africa (Spain, Maghreb, Cape Verde, and Chad), (ii) east Mediterranean (Israel), and (iii) southwest Asia (Iraq, Iran) (Table 1). Genetic variation was hierarchically partitioned into variation among defined population groups, among populations within groups, and among individuals within populations. The AMOVA was run in arlequin, testing significance with 10,000 permutations of the original data (Excoffier & Lischer, 2010).

### 2.4 Analyses of the ddRAD‐seq data

#### 2.4.1 Genetic diversity

We used the program *populations* from stacks to calculate observed (*H*_O_) and expected heterozygosity (*H*_E_), nucleotide diversity (*π*), and Wright's inbreeding coefficients (*F*_IS_) for each genotyped population, considering either all positions (polymorphic and nonpolymorphic) or only considering variant (polymorphic) positions. We also calculated individual heterozygosity, estimated as the proportion of heterozygous loci for each sample (*H*_O_) among our SNP dataset (i.e., excluding nonpolymorphic positions). Levene's tests showed that variances in individual heterozygosity were not homogeneous across populations (see Section 3.2.2). For this reason, we analyzed differences among populations in individual heterozygosity using nonparametric tests (i.e., Kruskal–Wallis *H*‐tests and Mann–Whitney *U*‐tests).

#### 2.4.2 Genetic differentiation and structure

First, we used arlequin to calculate genetic differentiation (*F*_ST_) among wild populations and between wild populations and the captive populations putatively derived from them, determining statistical significance with Fisher's exact tests after 10,000 permutations. Second, we quantified genetic structure and admixture among genotyped individuals using the Bayesian–Markov chain Monte Carlo clustering method implemented in the program structure v. 2.3.3 (Pritchard et al., 2000). We ran structure analyses for all genotyped individuals jointly (i.e., wild and captive populations) and for only the wild populations from Spain and Iraq (Table 1). We ran the analyses assuming correlated allele frequencies and admixture and without using prior population information (Hubisz et al., 2009). We conducted 15 independent runs for each value of *K* (from *K* = 1 to *K* = 8) to estimate the most likely number of genetic clusters with 200,000 MCMC cycles, following a burn‐in step of 100,000 iterations. We retained the 10 runs having the highest likelihood for each value of *K* and determined the number of genetic clusters that best describes our data according to log probabilities of the data (LnPr(X|*K*)) (Pritchard et al., 2000) and the Δ*K* method (Evanno et al., 2005), as implemented in structure harvester (Earl & vonHoldt, 2012). We used clumpp v. 1.1.2 and the Greedy algorithm to align multiple runs of structure for the same *K* value (Jakobsson & Rosenberg, 2007) and distruct v. 1.1 (Rosenberg, 2004) to visualize the individuals´ probabilities of population membership in bar plots. To complement the above, we performed principal component analysis (PCA) as implemented in the r package “adegenet” (Jombart, 2008) and obtained a co‐ancestry matrix in fineradstructure v. 0.3.2 (Malinsky et al., 2018). Before running PCAs, we replaced missing data by the mean allele frequency of the corresponding locus estimated across all samples (Jombart, 2008). As fineradstructure requires haplotype linkage information, we deselected the *write‐random‐snp* option in stacks (see Section 2.2.2) to export a dataset including all linked SNPs within each RAD locus. We ran fineradstructure following the software pipeline with default settings and used the r scripts *fineRADstructurePlot.R* and *FinestructureLibrary.R* available on GitHub (https://github.com/millanek/fineRADstructure; accessed at 06/11/2023) to plot the co‐ancestry matrix as a heatmap (Malinsky et al., 2018).

### 2.5 Testing alternative demographic models

**FIGURE 1:**
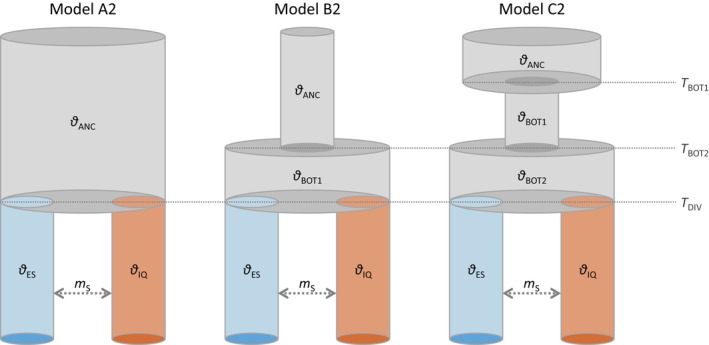
Demographic models tested using fastsimcoal2 for wild populations of marbled teal (*M. angustirostris*) from Spain (ES, in blue) and Iraq (IQ, in brown). Parameters include mutation‐scaled ancestral (*θ*_ANC_, *θ*_BOT1_, and *θ*_BOT2_) and contemporary (*θ*_ES_ and *θ*_IQ_) effective population sizes, migration rates per generation (*m*_S_), timing of divergence (*T*_DIV_), and timing of population size change (*T*_BOT1_ and *T*_BOT2_). The full set of tested models (i.e., considering alternative gene flow scenarios) is illustrated in Figure S1.

We used genomic data and the coalescent‐based approach implemented in fastsimcoal2 (Excoffier et al., 2013) to test alternative demographic and gene flow models, considering two demes corresponding to wild populations of marbled teal from Spain and Iraq (Figure 1). Pilot fastsimcoal2 analyses and reconstructions of effective population size (*N*_e_) through time in stairway plot indicate that major demographic changes experienced by the species pre‐date the split of the populations from Spain and Iraq, which have maintained a stable *N*_e_ since their divergence took place (see Section 3.4). For this reason, we tested three alternative demographic models considering that the ancestral population had a constant population size (i.e., one‐epoch model; Model A) or has experienced one (i.e., two‐epoch model; Model B) or two (i.e., three‐epoch model; Model C) population size changes (Figure 1). These three baseline demographic models were tested fitting different migration matrices connecting the populations from Spain and Iraq, including a scenario of strict isolation (SI) (i.e., total lack of post‐divergence gene flow) and an isolation‐with‐migration (IM) scenario considering that gene flow between the two demes was either symmetric (IM_S_) or asymmetric (IM_A_). The full set of tested models is illustrated in Figure S1.

We estimated the composite likelihood of the observed data given a specified model using the site frequency spectrum (SFS) and the simulation‐based approach implemented in fastsimcoal2. We calculated the folded joint SFS considering a single SNP per locus to avoid the effects of linkage disequilibrium. Because we did not include invariable sites in the SFS, we fixed the effective population size for the deme corresponding to the population from Iraq (*θ*_IQ_) to enable the estimation of other parameters in fastsimcoal2 (Excoffier et al., 2013). The effective population size fixed in the model was calculated from the level of nucleotide diversity (π) and the mutation rate per site per generation (μ = 4.83 × 10^−9^) estimated for other Anatidae (Lavretsky et al., 2019). Nucleotide diversity (π) was estimated from polymorphic and nonpolymorphic loci using stacks (Table 1). As the SFS is highly sensitive to the removal of rare variants, we obtained a dataset without a MAF filtering (see Section 2.2.2). We maximized the number of retained SNPs only exporting data for the specific populations involved in the calculation of the SFS (i.e., wild populations from Spain and Iraq). To remove all missing data for the calculation of the joint SFS, minimize errors in allele frequency estimates and maximize the number of SNPs retained, each population group was downsampled to 50% of individuals (i.e., 12 and 6 individuals for populations from Spain and Iraq, respectively; Table 1) using a custom Python script written by Andréa T. Thomaz and available on GitHub (https://github.com/ichthya/ThomazKnowles2020_scripts; accessed at 20/03/2023) (Thomaz & Knowles, 2020). Each model was run 100 replicated times considering 100,000–250,000 simulations for the calculation of the composite likelihood, 10–40 expectation‐conditional maximization (ECM) cycles, and a stopping criterion of 0.001 (Excoffier et al., 2013). We used an information‐theoretic model selection approach based on the Akaike's information criterion (AIC) to compare the set of candidate models and identify the one that is best supported by the data (Burnham & Anderson, 2002). After the maximum likelihood was estimated for each model in every replicate, we calculated the AIC scores as detailed in Thome and Carstens (2016). AIC values for each model were rescaled (ΔAIC) calculating the difference between the AIC value of each model and the minimum AIC obtained among all competing models (i.e., the best model has ΔAIC = 0). Point estimates of the different demographic parameters for the best supported model were selected from the run with the highest maximum composite likelihood. Finally, we calculated confidence intervals (based on the percentile method; e.g., de Manuel et al., 2016) of parameter estimates from 100 parametric bootstrap replicates by simulating SFS from the maximum composite likelihood estimates and re‐estimating parameters each time (Excoffier et al., 2013).

### 2.6 Reconstructing changes of *N*_e_ through time

We reconstructed changes in effective population size (*N*_e_) through time for wild and captive populations of marbled teal using stairway plot v. 2.1, a program that implements a flexible multiepoch demographic model based on the SFS that does not require whole‐genome sequence data or a predefined model for estimating past demographic histories (Liu & Fu, 2015, 2020). We used the same approach described for fastsimcoal2 analyses to compute the SFS for wild populations from Spain and Iraq, captive populations from Spain involved in reinforcement programs, and European zoos. We ran stairway plot considering the 4‐year generation time estimated for the species (BirdLife International, 2021) and other Anatidae (Lavretsky et al., 2019), assuming a mutation rate of 4.83 × 10^−9^ per site per generation (Lavretsky et al., 2019), and performing 200 bootstrap replicates to estimate 95% confidence intervals.

## 3 RESULTS

### 3.1 mtDNA data

#### 3.1.1 Haplotype composition of populations

**FIGURE 2:**
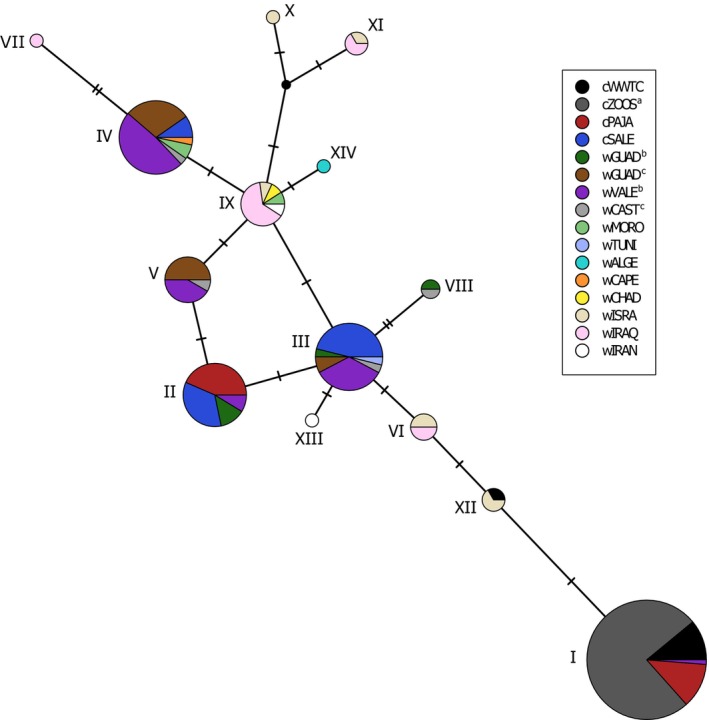
Haplotype network (TCS algorithm) based on mtDNA control region sequences for captive and wild populations of marbled teal (*M. angustirostris*). Circle size is proportional to the number of samples with each haplotype (see Table S1), and mutational steps are marked with hashes. ^a^All zoos from continental Europe present the same haplotype (haplotype I) and were grouped under the code cZOOS; ^b^Contemporary samples from Spain (wGUAD: 2005–2020; wVALE: 1991–2020); ^c^Historical samples from Spain predating the massive release of captive‐bred birds under reinforcement programs (wGUAD: 1967–1978; wCAST: 1970). All other population codes as described in Table 1.

We found 14 haplotypes for the sequenced fragment of the mtDNA control region across all analyzed populations (*n* = 201 sequences; Table S1; Figure 2). All contemporary individuals from European zoos carried the same haplotype (haplotype I, Table S1; Figures 2 and 4c). The museum specimen corresponding to one of the founders of the captive population of the WWT had a haplotype also recovered in two historical specimens from Israel (haplotype XII; Table S1), with only one nucleotide difference from haplotype I (Figure 2). Surprisingly, haplotypes I and XII were not found in any contemporary wild individual from Iraq (Table S1), the documented source of the birds used to establish the captive population at WWT from which, in turn, the rest of European zoos were founded. Note, however, that haplotype VI retrieved from seven birds from Iraq is separated from haplotypes I and XII by only one and two nucleotide differences, respectively (Figure 2). Haplotype I was present in 50% of the genotyped individuals from Cañada de los Pájaros (cPAJA), one of the Spanish centers currently involved in ex situ conservation programs (Table S1; Figures 2 and 4c). Haplotype I was found in one contemporary wild bird from Valencia region, but was not recovered in any historical sample from Spain (Table S1), which suggests recent genetic introgression from European zoo populations after the release of captive‐bred birds in the context of reinforcement programs. In Spain, six haplotypes were found among contemporary individuals (five when excluding haplotype I), and four among historical specimens, which were all found in the contemporary sample (Table S1). The other haplotypes (II, III, and IV) found in the populations from the two centers involved in captive breeding programs were also recovered at high frequencies in the contemporary wild population from Spain (Table S1; Figures 2 and 4c). Focusing exclusively on wild populations, nine haplotypes were private to a single country (I, II, V, VII, VIII, X, XII, XIII, and XIV) and five were shared by two (III, VI, and XI), three (IV) or five countries (IX) (Table S1; Figure 2). Only haplotype IX was shared among western Mediterranean and west Africa (wMORO and wCHAD), east Mediterranean (wISRA), and southwest Asia (wIRAQ, wIRAN) biogeographical populations (sensu Scott & Rose, 1996; Table S1).

#### 3.1.2 Genetic diversity

Genetic diversity statistics for the mtDNA dataset (*H*, Hd, and π) are presented in Table 1. In Spain, contemporary wild samples had significantly higher levels of genetic diversity than historical samples predating reinforcement programs (Welch's *t*‐test in all cases; Hd: *t* = 4.59, *p* < 0.001; π: *t* = 2.59, *p* = 0.015). However, when the only wild bird from Spain carrying haplotype I (i.e., putatively introgressed from the European zoo captive population; see Section 3.1.1) was excluded from the analyses, differences between contemporary and historical samples only remained statistically significant for Hd (Hd: *t* = 3.65, *p* = 0.001; π: *t* = 0.50, *p* = 0.622).

The contemporary wild population from Spain had significantly higher levels of Hd than the contemporary wild population from Iraq (*t* = 2.38, *p* = 0.036), but estimates of π for the two populations were virtually identical (*t* = 0.00, *p* = 1.000). However, when the wild specimen from Spain carrying haplotype I was excluded from the analyses, genetic diversity of wild populations from Spain and Iraq did not significantly differ for either Hd (*t* = 2.00, *p* = 0.071) or π (*t* = 0.92, *p* = 0.377).

The European zoo population (pooling individuals from the different centers in Table 1) had a single haplotype and, thus, presented significantly lower levels of genetic diversity than its putative wild source population from Iraq (Hd: *t* = 16.98, *p* < 0.001; π: *t* = 12.82, *p* < 0.001). Captive populations from Spain involved in ex situ conservation programs (cPAJA and cSALE) had significantly lower levels of genetic diversity than the contemporary wild population from Spain in analyses either including the wild bird with haplotype I (cPAJA, Hd: *t* = 20.24, *p* < 0.001; π: *t* = 19.60, *p* < 0.001; cSALE, Hd: *t* = 8.53, *p* < 0.001; π: *t* = 14.19, *p* < 0.001) or excluding it (cPAJA, Hd: *t* = 18.73, *p* < 0.001; π: *t* = 18.72, *p* < 0.001; cSALE, Hd: *t* = 7.51, *p* < 0.001; π: *t* = 12.50, *p* < 0.001). Tajima's tests were not statistically significant for any historical or contemporary wild population (*p* > 0.100), suggesting that the species has not experienced recent demographic changes.

#### 3.1.3 Genetic differentiation and structure

Contemporary and historical wild populations from Spain were not genetically differentiated (*Φ*_ST_ = 0.013, *p* = 0.241). The contemporary wild populations from Spain and Iraq were significantly differentiated (*Φ*_ST_ = 0.156 and *p* = 0.040; for comparisons between all pairs of wild populations, see Table S2a). Populations from Spain and Israel were also significantly differentiated, but there was no statistically significant difference between Iraq and Israel (i.e., between south‐west Asia and east Mediterranean, Table S2).

The European zoo population, which exclusively carried haplotype I, was genetically differentiated from its putative wild source population from Iraq (*Φ*_ST_ = 0.939 and *p* < 0.001). Likewise, captive populations from the two ex situ Spanish centers were genetically differentiated from the contemporary wild population from Spain (cPAJA: *Φ*_ST_ = 0.482, *p* < 0.001; cSALE: *Φ*_ST_ = 0.126, *p* = 0.007). Analogous analyses excluding the contemporary wild bird from Spain carrying haplotype I provided qualitatively similar results. AMOVA analyses showed that a high percentage of variation was attributed to differences among individuals within populations and, to a lesser extent, to differences among the three biogeographical populations (Scott & Rose, 1996) (Table 2). However, differentiation among populations within groups explained a very small percentage of variation and was not statistically significant (Table 2). AMOVAs run for datasets including all wild individuals and excluding contemporary samples from Spain yielded qualitatively similar results, although the percentage of variation explained by differences among groups was slightly higher when contemporary samples from Spain were excluded (Table 2). The haplotype network showed a lack of phylogeographic structure, with the main biogeographical regions sharing haplotypes with each other and not forming monophyletic groups (Figure 2; Table S1; see also Section 3.1.1).

**TABLE 2:** Results of AMOVAs for wild populations of marbled teal (*M. angustirostris*).

	Sum of squares	Variance components	Percentage of variation	*P*‐value
(A) All samples				
Among groups	8.20	0.20	19.89	0.024
Among populations within groups	5.79	−0.02	−2.12	0.585
Among individuals within populations	63.47	0.85	82.23	0.009
(B) Excluding contemporary samples from Spain				
Among groups	7.29	0.21	20.54	0.012
Among populations within groups	5.51	−0.02	−1.67	0.338
Among individuals within populations	32.63	0.84	81.14	0.012

*Note*: Analyses are based on mtDNA control region sequences and were performed considering the three biogeographical populations delineated by Scott and Rose (1996), namely (i) western Mediterranean and west Africa (ES, MA, TN, DZ, CV, and TD), (ii) east Mediterranean (IL), and (iii) south‐west Asia (IQ and IR). ISO country codes for samples included in each group are indicated in parentheses (see Table 1). AMOVAs were run for datasets (a) including all analyzed individuals and (b) excluding contemporary samples (1991–2020) from Spain.

### 3.2 ddRAD‐seq data

#### 3.2.1 Genomic dataset

**FIGURE 3:**
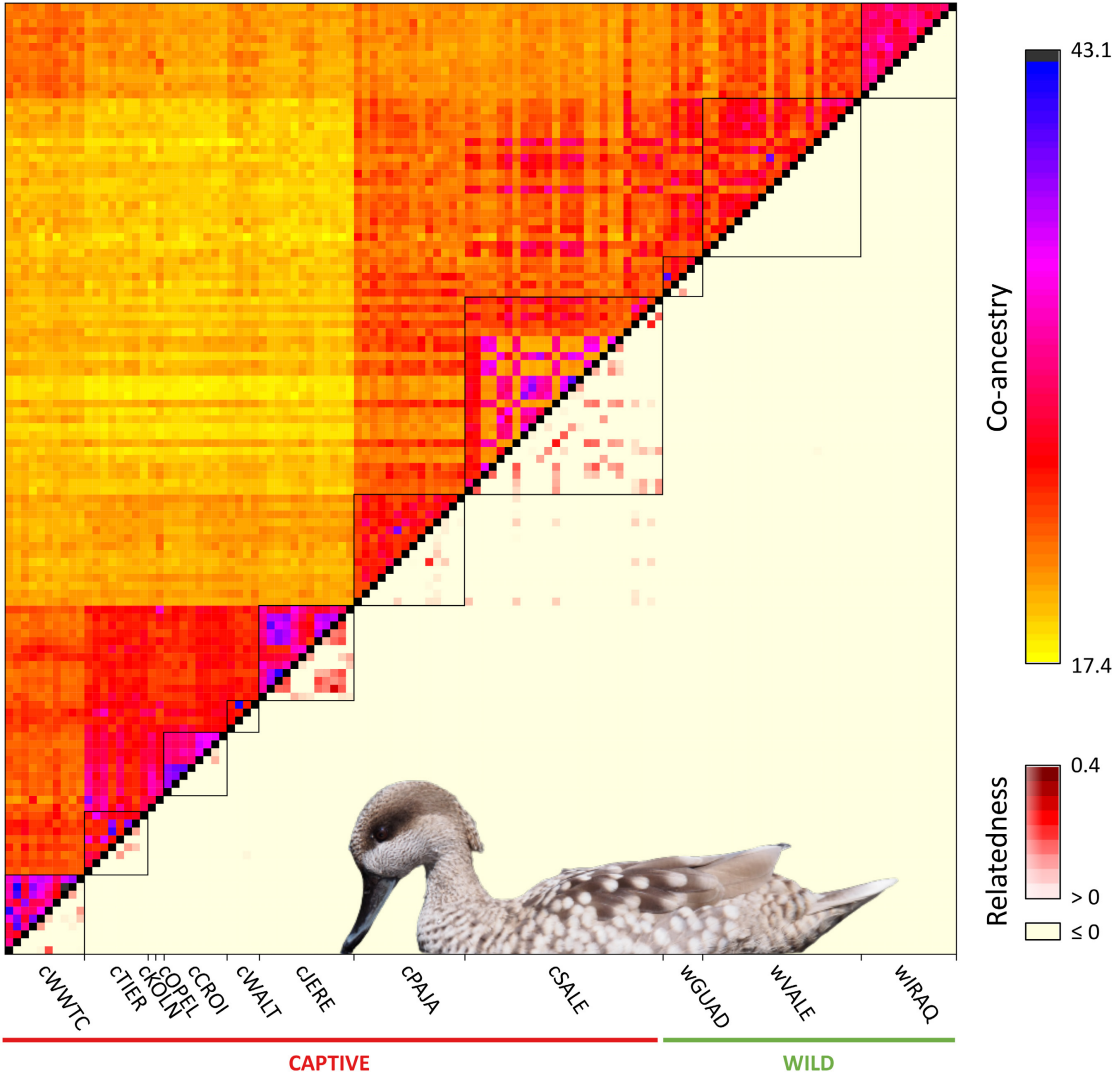
Heatmap showing co‐ancestry (above the diagonal) and relatedness (below the diagonal) values between each pair of genotyped individuals of marbled teal (*M. angustirostris*). Simple co‐ancestry was estimated with fineradstructure. Relatedness was calculated using the kinship coefficient (*φ*_ij_) developed by Manichaikul et al. (2010), which ranges from 0 (or negative values) for unrelated individuals to 0.5 for individual‐self. All relatedness values hidden by the inset picture are *φ*_*ij*_ ≤ 0 (i.e., light yellow). Picture of marbled teal by Francis C. Franklin (licensed under CC BY‐SA 2.0). Population codes as described in Table 1.

The average number of reads retained per individual after the different quality filtering steps was 6,101,389 (range = 231,802–20,327,805 reads). After filtering out loci with a MAF <0.01 and that were represented in <75% of individuals, the final dataset contained 2634 unlinked SNPs. Analyses of relatedness revealed that 147 pairs of individuals presented some degree of kinship (*φ*_ij_ > 0; Figure 3). As expected, most pairs of related individuals are from captive populations (*n* = 146 pairs) and involved individuals from the same population (Figure 3). The only comparisons involving individuals from different populations that showed a kinship relationship were 15 pairs of captive birds from cPAJA and cSALE, which provides evidence for the recent exchange of individuals between the two Spanish centers involved in ex situ conservation programs (Figure 3). Among wild populations, only two individuals from Guadalquivir Marshes (wGUAD) had a kinship relationship equivalent to a second‐ or third‐degree relative (*φ*_ij_ = 0.09; Figure 3; Manichaikul et al., 2010). For all the subsequent analyses, we retained only one individual (the one presenting a lower proportion of missing data) among those that shared some degree of kinship, in order to create a dataset excluding related individuals (*φ*_ij_ < 0). This dataset contained 75 individuals (for details, see Table 1).

#### 3.2.2 Genetic diversity

**FIGURE 4:**
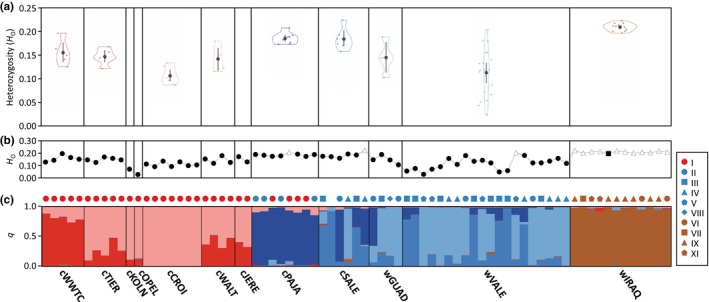
Genetic diversity (a, b) and structure (c) for captive and wild populations of marbled teal (*M. angustirostris*). (a) Violin plots show estimates of heterozygosity (*H*_O_) for each individual (small coloured dots) and mean and confidence intervals (black dots and vertical bars, respectively) for each population (*n* ≥ 4). (b) Heterozygosity (*H*_O_) for each individual, with black dots and white triangles indicating estimates below and above the minimum value (black square) observed across all samples from Iraq, respectively. (c) Genetic assignments based on the program structure for *K* = 6. Each individual is represented by a vertical bar partitioned into *K* coloured segments showing the individual's probability (*q*) of belonging to the cluster with that colour. Symbols on top of the structure bar plot indicate the haplotype of each individual based on mtDNA control region sequences (legend on the right); note this information is missing for one individual from cSALE. Analyses are based on a dataset only including unrelated individuals (*φ*_*ij*_ ≤ 0; see Figure 3). Vertical black lines separate different populations. Population codes as described in Table 1.

Genetic diversity statistics for the ddRAD‐seq dataset (*π*, *H*_O_, *H*_E_, and *F*_IS_) calculated for all positions (polymorphic and nonpolymorphic) and only considering variant positions (polymorphic) are presented in Tables 1 and S3. Individual heterozygosity ranged from 0.023 to 0.224 (Figure 4a,b; see also Figure S3). Variance in individual heterozygosity differed among populations (Levene's tests in all cases; *F*_8,62_ = 4.31, *p* < 0.001; Figure 4a). The contemporary wild population from Spain had significantly higher variance in individual heterozygosity than the contemporary wild population from Iraq (*F*_1,34_ = 14.51, *p* < 0.001). The European zoo population had significantly higher variance in individual heterozygosity than its wild source population from Iraq (*F*_1,35_ = 9.46, *p* = 0.004). However, variance in individual heterozygosity was higher in the wild population from Spain than in the two ex situ Spanish centers, although the difference was only statistically significant for cPAJA (cPAJA: *F*_1,30_ = 8.10, *p* = 0.008; cSALE: *F*_1,28_ = 3.18, *p* = 0.085). Individual heterozygosity differed among populations (Kruskal–Wallis *H*‐test; *H* = 47.94, df = 8, *p* < 0.001). The population from Iraq had the highest levels of individual genetic diversity, with only three individuals from either captive or wild European populations presenting levels of heterozygosity above the minimum value observed across all samples from Iraq (Figure 4b). Accordingly, individual heterozygosity was significantly lower in the wild population from Spain than the wild population from Iraq (Mann–Whitney *U*‐tests in all cases; *U* = 3, *N*_1_ = 24, *N*_2_ = 12, *p* < 0.001). Similarly, the European zoo population had significantly lower levels of individual heterozygosity than its wild source population from Iraq (*U* = 0, *N*_1_ = 25, *N*_2_ = 12, *p* < 0.001). However, individual heterozygosity was significantly lower in individuals from the wild population from Spain than in individuals from the two Spanish centers involved in ex situ conservation programs (cPAJA: *U* = 18, *N*_1_ = 8, *N*_2_ = 24, *p* < 0.001; cSALE: *U* = 15, *N*_1_ = 6, *N*_2_ = 24, *p* = 0.003). We found no statistically significant differences in genetic diversity among individuals assigned (structure *q*‐value >0.8) to the two main genetic clusters inferred by structure (i.e., light blue and medium dark blue clusters in Figure 4c) within the wild population from Spain (Mann–Whitney *U* tests in all cases; *U* = 44, *N*_1_ = 4, *N*_2_ = 14, *p* < 0.001; see Section 3.2.3 for analyses of the genetic structure). For comparisons between all pairs of populations, see Tables S4 and S5. The considerable variance in genetic diversity (range: 0.02–0.20; Figure 4a), together with significant genetic substructure (i.e., Wahlund effect; Figure 4c; see Section 3.2.3), resulted in the wild population from Spain having estimates for the inbreeding coefficient (*F*_IS_) that were up to seven times higher than for other study populations (Table 1; Table S3).

#### 3.2.3 Genetic differentiation and structure

Genetic differentiation (*F*_ST_) between wild populations from Spain and Iraq was low but significantly different from zero (*F*_ST_ = 0.058, *p* < 0.001; for comparisons between all pairs of wild populations, see Table S2b). The European zoo population was genetically differentiated from its putative wild source population from Iraq (*F*_ST_ = 0.117, *p* < 0.001), and similar results were obtained when comparisons only included the captive population from the WWT (*F*_ST_ = 0.112, *p* < 0.001) or zoo populations from continental Europe (*F*_ST_ = 0.142, *p* < 0.001). When compared with the two centers currently involved in ex situ conservation programs, the contemporary wild population from Spain was genetically differentiated from cPAJA (*F*_ST_ = 0.082, *p* < 0.001), but not from cSALE (*F*_ST_ = 0.003, *p* = 0.791).

structure analyses (2634 unlinked SNPs) including all individuals identified the most likely number of genetic clusters as *K* = 2 according to the Δ*K* criterion, but LnPr(X|*K*) reached a plateau at *K* = 6 (Figure S3a). For *K* = 2, individuals from European zoos separated from the individuals from the other populations, albeit several populations presented a considerable degree of admixed ancestry (cWWTC, cPAJA, and wIRAQ; Figure S4a). For *K* = 6, two of the genetic clusters were mostly represented in individuals from zoo populations (cWWTC, cTIER, cKOLN, cOPEL, cWALT, and cJERE). The wild individuals from Spain (wGUAD and wVALE) and those from the captive populations for reinforcement programs (cPAJA and cSALE) were split into three genetic clusters, and the final cluster corresponded to the wild individuals from Iraq (wIRAQ; Figure 4c). Among zoo populations, one cluster was mostly represented in captive‐bred birds from the WWT and the other in zoo populations from continental Europe (Figure 4c; see also Figure S5); a large proportion of shared ancestry among most zoo populations suggests incomplete genetic differentiation (i.e., retained ancestry). Individuals from Spain, whether wild or captive‐bred for ex situ programs, split into three genetic clusters. Whereas one of these clusters was mostly represented by individuals from the captive population cPAJA, the other two did not show a clear correspondence with a particular captive or wild population, and individuals presented different levels of admixture among them (Figure 3c). Remarkably, six birds from cPAJA and three birds from cSALE presented genetic admixture (3%–16%) with zoo populations, especially with the genetic cluster (2%–13%) predominantly represented in the breeding stock of zoos from continental Europe (Figure 4c). Wild birds from Spain showed no signatures of admixture with the European zoo stock (Figure 4c); note, however, that the only wild bird from Spain carrying haplotype I (see Section 3.1.1) was a museum specimen from 1992 and, thus, it could not be genotyped for ddRAD‐seq. Results for other *K* values are presented in Figure S4a.

structure analyses based on wild individuals only indicated that the most likely number of clusters was *K* = 3 according to the Δ*K* criterion, but LnPr(X|*K*) steadily increased until *K* = 8 (Figure S3b). Analyses for the different *K* values showed a clear separation of individuals from Spain and Iraq in two genetic clusters with negligible genetic admixture. For *K* = 3–4, wild individuals from Spain were assigned to different genetic clusters that in most cases were only represented by one or a few individuals, showed little correspondence with sampling localities, and often presented considerable genetic admixture (Figure S4b).

**FIGURE 5:**
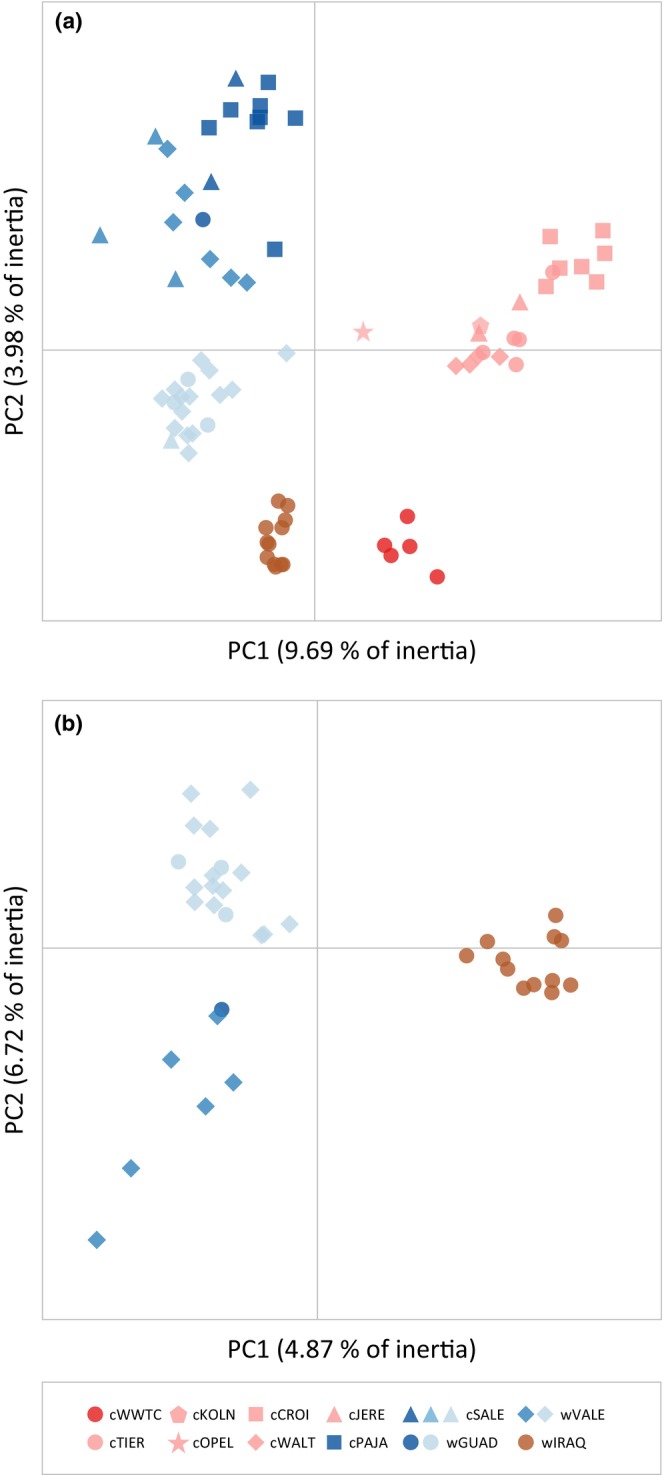
PCAs of genetic variation for captive and wild populations of marbled teal (*M. angustirostris*). Analyses are based on datasets only including unrelated individuals (*φ*_*ij*_ ≤ 0; see Figure 3) and were run for all populations (a) and separately for wild populations (b). Colours indicate the main genetic cluster to which each individual was assigned according to structure analyses for *K* = 6 (see Figure 4c). Population codes as described in Table 1.

PCA (2634 unlinked SNPs), run either for all genotyped populations or separately for wild populations, yielded results in line with those obtained for Bayesian clustering analyses in structure (Figure 5). According to PCAs, individuals from most populations were grouped in tight genetic clusters (Figure 5). The only exception were the wild populations from Spain (wGUAD and wVALE) and the captive‐bred population from one of the Spanish centers involved in reinforcement programs (cSALE), which presented considerable dispersion and evidence of genetic substructure in line with the results of structure at different hierarchical levels (Figures 4 and S4). Finally, fineradstructure analyses (4830 SNPs) confirmed the results from structure and PCA analyses, showing co‐ancestry values congruent with the probability of assignment of individuals to the genetic clusters identified by structure at the different hierarchical levels (Figures 3 and S5).

### 3.3 Testing alternative models of divergence

The joint SFS used for fastsimcoal2 analyses contained 2408 unlinked SNPs. the most supported demographic model (Model A2) was the one fitting a constant effective population size (*N*_e_) for the ancestral population and an isolation‐with‐migration scenario of divergence of populations from Spain and Iraq, with symmetric gene flow between the two demes (Table 3; Figure 1). Alternative models provided a much poorer fit to our genomic data and were statistically highly unlikely (ΔAIC > 11; Table 3). Parameter estimates under the most supported model indicate that the two populations split 407 generations ago from an ancestral population, with a *N*_e_ much smaller (3225 haploid individuals) than that sustained by contemporary populations of the species from either Spain (16,388 haploid individuals) or Iraq (48,654 haploid individuals) (Table 4). Considering a 4‐year generation time for the species, the split between Spanish and Iraqi populations was estimated to take place ~1628 years ago (95% CI = 1800–8436 years ago; Table 4). Note that parametric confidence intervals do not necessarily include the point estimate (Table 4).

**TABLE 3:** Alternative demographic models tested using fastsimcoal2 for wild populations of marbled teal (*M. angustirostris*) from Spain and Iraq, with the best supported scenario (ΔAIC = 0) highlighted in bold.

Model	Demography	Divergence	Migration	lnL	*K*	AIC	ΔAIC	*ω*_*i*_
A1	One‐epoch	SI	—	−3777.76	3	7561.51	14.48	0.00
**A2**	**One‐epoch**	**IM**	**Symmetric**	**−3769.52**	**4**	**7547.04**	**0.00**	**0.99**
A3	One‐epoch	IM	Asymmetric	−3774.05	5	7558.10	11.06	0.00
B1	Two‐epoch	SI	—	−3776.73	5	7563.46	16.42	0.00
B2	Two‐epoch	IM	Symmetric	−3776.78	6	7565.57	18.53	0.00
B3	Two‐epoch	IM	Asymmetric	−3777.48	7	7568.96	21.93	0.00
C1	Three‐epoch	SI	—	−3777.96	7	7569.91	22.88	0.00
C2	Three‐epoch	IM	Symmetric	−3776.91	8	7569.81	22.78	0.00
C3	Three‐epoch	IM	Asymmetric	−3777.55	9	7573.10	26.06	0.00

*Note*: Models (illustrated in Figure S1) consider different demographic scenarios for the ancestral population (single‐, two‐ or three‐epoch models) and alternative scenarios of divergence (SI: strict isolation; IM: isolation‐with‐migration, considering either symmetric or asymmetric gene flow).

Abbreviations: ∆AIC, difference in AIC value from that of the strongest model; AIC, Akaike's information criterion value; *k*, number of parameters in the model; lnL, maximum likelihood value of the model; ω_*i*_, AIC weight.

**TABLE 4:** Parameters inferred from coalescent simulations with fastsimcoal2 under the most likely demographic model (Model A2, illustrated in Figure 1) for wild populations of marbled teal (*M. angustirostris*) from Spain (ES) and Iraq (IQ).

Parameter	Point estimate	Lower bound	Upper bound
*θ*_ANC_	3225	950	3829
*θ*_ES_	16,388	16,770	58,625
*θ*_IQ_	48,654	—	—
*T*_DIV_	407	450	2109
*m*_S_	8.71 × 10^−7^	1.32 × 10^−6^	5.02 × 10^−4^

*Note*: The table shows point estimates and lower and upper 95% confidence intervals for each parameter, which include mutation‐scaled ancestral (*θ*_ANC_) and contemporary (*θ*_ES_ and *θ*_IQ_) effective population sizes, historical migration rates per generation (*m*_S_), and timing of divergence (*T*_DIV_), with time given in units of generations. Contemporary effective population size of the deme corresponding to populations from Iraq (*θ*_IQ_) was calculated from its levels of nucleotide diversity (π) and fixed in fastsimcoal2 analyses to enable the estimation of all other parameters (see Section 2.5 for further details).

### 3.4 Reconstructing changes of *N*_e_ through time

**FIGURE 6:**
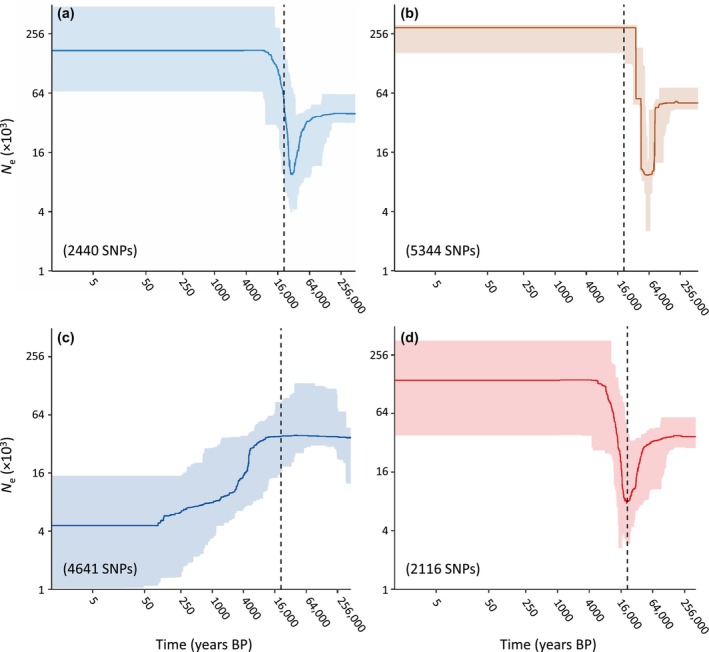
Demographic history of wild and captive populations of marbled teal (*M. angustirostris*) inferred using stairway plot. Analyses were performed for wild populations from Spain (a) and Iraq (b), captive populations from Spain involved in reinforcement programs (c), and European zoos (d). Panels show median (solid lines) and 2.5 and 97.5 percentiles (shaded areas) of effective population size (*N*_e_) through time, estimated assuming a mutation rate per site per generation of 4.83 × 10^−9^ and 4‐year generation time for the species (both axes in a logarithmic scale). The number of unlinked SNPs used to calculate the SFS in each analysis is indicated in parentheses. Vertical dashed lines indicate the last glacial maximum (LGM, ~ 21 ka BP).

stairway plot analyses showed that wild populations of marbled teal from Spain and Iraq and captive populations from European zoos have experienced parallel demographic trajectories (Figure 6a–c). These populations went through a marked demographic bottleneck during the last glacial period, which resulted in a reduction of effective population size (*N*_e_) of ~75% with respect to levels sustained by pre‐bottleneck ancestral populations (Figure 6). After the last glacial period, these populations experienced sudden demographic expansions followed by a stable *N*_e_ until the present day (Figure 6). Remarkably, such demographic expansion led to estimates of *N*_e_ that largely surpassed that of pre‐bottleneck populations, which has resulted in contemporary populations sustaining a *N*_e_ ~5 times larger than their respective ancestral populations (Figure 6). In contrast, stairway plot analyses for captive populations from Spain involved in reinforcement programs (cPAJA and cSALE) suggest a continuous decline of *N*_e_ from the last glacial period to the present (Figure 6d). This result must be interpreted with caution, considering the marked genetic heterogeneity of the samples from the two ex situ conservation centers and their genetic introgression from the European zoo stock (see Section 3.2.3 and Figure 3c).

## 4 DISCUSSION

Genetic data obtained for both contemporary and museum specimens showed that range‐wide populations of marbled teal diverged recently (<8000 years) and present a lack of phylogeographic structure. However, restricted gene flow among main biogeographical units (i.e., flyways) has likely contributed to their genotypic and haplotypic distinctiveness. Genomic‐based reconstructions of historical changes in *N*_e_ through time showed that wild populations from Spain and Iraq have experienced parallel demographic trajectories, undergoing marked declines during the last glacial period followed by sudden demographic expansions and stability until the present day. Although high estimates of inbreeding in contemporary populations from Spain indicate negative consequences of the continuous reduction and fragmentation of Western Mediterranean populations during the past century, our genomic data did not provide evidence that such processes have been accompanied by recent genetic bottlenecks compatible with human‐driven declines of the species. Finally, genetic assessment of captive‐bred populations from two Spanish centers involved in ex situ conservation programs showed they were predominantly established with native birds, but present some degree of admixed ancestry with European zoo populations, which themselves were founded with birds originating from the genotypically distinct population of Iraq. However, despite the massive release of captive‐bred birds since the 1990s, our data indicate that contemporary wild populations from Spain have experienced very limited introgression from this European zoo stock.

### 4.1 Genetic structure of wild populations

Mitochondrial DNA data retrieved from both museum and contemporary specimens revealed a weak phylogeographic structure across the distribution range of the species. The main biogeographic regions did not form monophyletic groups, but instead shared multiple haplotypes (Figure 2; Table S1). Accordingly, coalescent analyses based on genomic data estimated that wild populations from Spain and Iraq diverged recently, most likely during the Holocene (<8000 years ago; Table 3). These results are in line with the lack of phylogeographic structure found in the co‐distributed white‐headed duck (*Oxyura leucocephala*; Muñoz‐Fuentes et al., 2005, 2008) and limited genetic differentiation among conspecific populations of many other Anatidae (e.g., Kraus et al., 2011, 2013; Seibert et al., 2023). However, although a model of divergence with symmetric gene flow (i.e., isolation‐with‐migration) was the one providing a better fit to our genomic data, estimates of migration rates per generation between the wild populations from Spain and Iraq were low (Table 3). This, together with relatively small *N*_e_ and historical population fragmentation, can explain the quick development (<2000 generations) of genetic structure and significant differentiation among the main biogeographical populations (i.e., flyways) for both mtDNA and nuclear genomic markers, the negligible admixed ancestry between wild populations from Spain and Iraq, and the high probability of membership to their respective genetic clusters (Figure 4; Tables 2 and S2). Given the genetic differentiation between populations from Spain and Iraq was low in absolute terms for both nuclear (*F*_ST_ = 0.058) and mtDNA (*Φ*_ST_ = 0.156) markers, and the rapid genetic drift that was evident in the captive populations, we advocate a considered approach to future ex situ and in situ conservation efforts. Both the adaptive potential of the populations and potential differences corresponding to the West Mediterranean and south‐west Asia flyways (Scott & Rose, 1996) need to be considered in future conservation strategies. Balancing the risks of inbreeding and outbreeding depression is vital and we suggest that an evaluation is conducted to identify areas for improvement in current breeding and release strategies (Frankham, 2015; Fraser & Bernatchez, 2001; Moritz, 2002; Weeks et al., 2011; e.g., Peters et al., 2016; for implications for ex situ conservation, see Section 4.3).

An intriguing finding is the genetic substructure characterizing wild samples from Spain that, in contrast with the tight genetic grouping and homogeneity of wild specimens from Iraq, are assigned to up to three distinct genotypic clusters with different levels of admixture and no correspondence with the two main sampling areas (i.e., Guadalquivir Marshes and Valencia region) or the different mtDNA haplotypes retrieved (Figure 4c and S4). Different nonexclusive factors could explain the genetic substructure observed among wild samples from Spain. One possibility is that some genotyped individuals are immigrants from other nearby breeding populations (e.g., Maghreb region; Green et al., 2002) that might belong to distinct genotypic clusters (e.g., Peters et al., 2016). Such a genetic substructure at a regional scale—also reported for other vagile vertebrate species at the verge of extinction such as the imperial eagle (*Aquila adalberti*; Martínez‐Cruz et al., 2007) and the Iberian lynx (*Lynx pardinus*; Casas‐Marce et al., 2013)—might have arisen as a consequence of the fragmentation of contemporary populations of the species in the Western Mediterranean (BirdLife International, 2022). Movements of birds between the Maghreb region and the Iberian Peninsula have been regularly reported in the literature (Green et al., 2004; Pérez‐García et al., in press) but philopatric behaviour (i.e., return to natal/breeding grounds; Blums et al., 2002) and/or poor performance of immigrants could potentially have resulted in a limited admixture among genetic pools and contributed to the maintenance of local substructure (Wang & Bradburd, 2014). Another possibility to explain the genotypic disequilibrium of wild Spanish populations is that the massive release of captive‐bred individuals has artificially contributed to the observed genetic substructure (e.g., Baratti et al., 2015; Champagnon et al., 2013; Söderquist et al., 2017). The two centers currently involved in ex situ conservation programs show a marked genetic structure, probably resulting from a limited number of founder birds, genetic drift over multiple generations of captive breeding, and different levels of genetic introgression from the European zoo stock. This could ultimately result in the release of cohorts of birds with different genetic backgrounds and assigned to distinctive genetic clusters (Figure 4c; see Section 4.3). Although the birds we sampled and genotyped were unringed at the time of sampling and, thus, they are expected to be wild‐born individuals, it is possible that some of them were descendants of the many captive‐bred birds released in Spain over recent decades. In the most recent years, the numbers of individuals released from captivity each year has even exceeded estimates of the remaining wild population (Fundación Biodiversidad, 2022; Pérez‐García et al., in press).

### 4.2 Demographic history and the genetic diversity of wild populations

Genomic‐based demographic reconstructions showed that wild populations from Spain and Iraq have experienced parallel demographic trajectories during the past 250,000 years, with a marked bottleneck during the last glacial period (~115,000–11,700 years ago) followed by a sudden expansion and subsequent demographic stagnation from the onset of the Holocene until the present day (Figure 6). These results are in line with inferences from our coalescent‐based analyses of population divergence, which suggested that the two populations diverged <8000 years ago from an ancestral population with an effective population size much smaller than that sustained by contemporary populations (Table 4). Thus, post‐bottleneck demographic expansions resulted in contemporary populations of the species presenting much larger effective population sizes than their most recent common ancestral population (Figure 6). Such demographic history is expected for thermophilous organisms, which have likely faced unfavourable environmental conditions and population contractions during the coldest stages of the Pleistocene and demographic expansions coinciding with warm interglacials. A rapid postglacial expansion has been suggested for the largely co‐distributed white‐headed duck based on mtDNA (Muñoz‐Fuentes et al., 2005) and analogous demographic profiles have also been inferred based on genomic data for other thermophilous organisms with similar distributional ranges such as the Moroccan locust (*Dociostaurus maroccanus*; González‐Serna et al., 2020) and the Eurasian wild pea (*Pisum sativum*; Hellwig et al., 2022).

Although the wild population from Spain presents lower levels of nuclear genetic diversity than the population from Iraq (Figure 4), demographic reconstructions and Tajima's *D* tests did not reveal any evidence of recent genetic bottlenecks (Figure 4). In the same line, we did not find a reduction of mtDNA genetic diversity in contemporary populations from Spain compared to historical museum specimens (Table 1). These results contrast with the lower levels of mtDNA genetic diversity found in the contemporary Spanish population of the white‐headed duck compared to historical samples, a species that has also undergone dramatic population declines during the past century (Muñoz‐Fuentes et al., 2005). In contrast to marbled teals, white‐headed ducks are more sedentary and there is no evidence of movement between Spain and the Maghreb (see Muñoz‐Fuentes et al., 2005 and references therein). These results suggest that the lower levels of genetic diversity in the wild marbled teal population from Spain compared to their south‐west Asia counterparts reflect historical differences in their respective effective population sizes (e.g., linked to the availability of suitable habitats and/or connectivity of local populations) rather than being a direct consequence of the more severe human‐driven decline experienced by western Mediterranean populations. However, the severe decline of census population size recorded in Mediterranean populations has likely imposed on them a “genetic debt” that may still take some generations to become payable and will probably result in a significant loss of genetic variation through time (e.g., Sovic et al., 2019). In this vein, historical demographic processes are unlikely to explain the higher levels of inbreeding—likely inflated by within‐population genetic substructure; Figure 4c—and variance of individual genetic diversity in wild populations from Spain compared to those from Iraq, a phenomenon that can be primarily attributed to contemporary mating among closely related individuals in fragmented and small populations (Figure 4b; Table 1). Albeit anecdotal, the detection of two wild birds from Guadalquivir Marshes with a kinship relationship equivalent to a second‐ or third‐degree relative (*φ*_ij_ = 0.09; Figure 3) is noteworthy, suggesting that the encounter and chances of mating between close relatives might be frequent in the free‐ranging population from Spain. Such reduced levels of genetic diversity in some individuals could in part explain the relatively frequent occurrence of leucistic plumage—lack of melanin pigmentation—recently reported in populations from Spain (see Figure S6; Salvador et al., 2023), a phenomenon that has often been associated to the effects of inbreeding in small and isolated bird populations and that would be the result of the expression of recessive alleles (Bensch et al., 2000; Holyoak, 1978). Beyond the expected higher heterogeneity in inbreeding levels due to the small size of remnant populations of marbled teal in the western Mediterranean, we cannot rule out the possibility that the release of captive‐bred individuals with contrasting levels of genetic diversity might have also contributed to increased variance in inbreeding of contemporary wild populations of the species (see also Section 4.1 for the genetic structure).

### 4.3 Genetic assessment of captive populations

Analyses of genetic structure showed that captive zoo populations separate into two genetic clusters, one mostly represented in centers from the WWT and another encompassing the remaining European zoos. These population groups showed considerable genetic admixture (~10%–20%; Figure 3c) and shared the mtDNA haplotype I, as expected since zoo populations in continental Europe were established from birds originated from the British stock, which in turn was founded in 1948 with only nine birds collected from Basra wetlands in Iraq (Hawkes, 1970; Jones, 1951). This very small number of founders has likely resulted in considerable genetic drift and loss of genetic diversity during >70 years of captive breeding, which can explain the lower levels of genetic diversity of zoo populations compared to their source wild population from Iraq (Figure 4a,b). Although the WWT population shows certain genetic affinity with the contemporary wild population from Iraq, genetic drift over multiple generations of captive breeding has probably resulted in the two populations segregating into distinct genetic clusters (Figures 4 and 5). Likewise, the only mtDNA haplotype carried by all European zoo populations was not retrieved from the contemporary wild population from Iraq, nor in any other contemporary or historical wild population, although haplotype VI from Iraq was separated by only two mutational steps from haplotype I of zoo populations (Figure 2). Thus, haplotype I might have gone extinct from contemporary populations or occur at low frequencies, explaining why it was not recovered among our limited number of samples from Iraq (*n* = 14; Table 1). These results are in line with the rapid genetic differentiation documented between native populations of ruddy duck (*Oxyura jamaicensis*) from North America and those established in Europe from individuals escaped from a captive population in WWT (UK), which was similarly founded by a very small number of birds (Muñoz‐Fuentes et al., 2006). Similarly to marbled teals, only one haplotype was found in the captive and introduced European populations of ruddy ducks but, in contrast, this haplotype was the most common in the North American source population (Muñoz‐Fuentes et al., 2006).

Genetic data showed a clear separation between captive populations from European zoos and those from the two Spanish centers (cSALE and cPAJA) involved in captive breeding for reinforcement. The latter clustered together with wild individuals from Spain, indicating the predominant native origin of birds used for ex situ conservation (Figures 4 and 5). It is noticeable, however, that 50% of the individuals from cPAJA had mtDNA haplotype I (Table S1; Figure 4c) and 70% birds from cPAJA and 50% from cSALE presented traces of genetic introgression (cPAJA: 4–16%; cSALE: 3%–8%) from European zoo populations (Figure 4c and S4), which corroborates genetic admixture between native and zoo genetic stocks in both centers. Admixture of different gene pools could explain the higher levels of genetic diversity of these captive populations compared to the contemporary wild population from Spain. The significant genetic differentiation between the wild population from Spain and both cPAJA (mtDNA and nuclear markers) and cSALE (mtDNA) could be explained by a combination of genetic drift (i.e., founder effects) and genetic introgression from European zoo genotypes into the two captive‐bred populations currently involved in ex situ conservation programs. The higher levels of genetic introgression from European zoo genotypes into cPAJA has likely resulted in stronger genetic differentiation between this population and its wild counterparts compared to cSALE (Figure 4c).

Despite massive releases in Spain of captive‐bred birds originating from these two centers (>2000 released birds in mainland Spain since 2009) and different European zoos (>190 released birds in Balearic Islands since 2015) (Fundación Biodiversidad, 2022), we found no evidence for genetic admixture between native and European zoo genotypes in the wild population of Spain (Figure 4), and haplotype I was only retrieved in a single wild bird from the Valencia region (Table S1). Importantly, this haplotype was not recovered in any other historical or contemporary wild population (Figure 2), which suggests recent genetic introgression from European zoo populations after the release of captive‐bred birds (Table S1). This limited introgression from European zoo genotypes into the contemporary wild population from Spain, despite the massive release of birds during the past three decades, suggests poor breeding performance or reduced survival of captive‐bred individuals in the wild, selection against introgressed individuals (i.e., maladapted genotypes and/or outbreeding depression; Wang & Bradburd, 2014), and/or a progressive dilution of alien genotypes after successive backcrosses with the native genetic stock that predominates in wild populations (i.e., genetic and demographic swamping; Todesco et al., 2016). Strong differences in behaviour between captive‐bred and wild individuals (e.g., lack of exposure to natural stimuli, use of food resources, movement pattern, and/or differences in social structure) could make it difficult for captive‐bred birds to successfully integrate and mate with wild populations (Crates et al., 2023). Accordingly, poor performance of captive‐bred individuals after their release into the wild has been well documented in mallards (Champagnon, Elmberg, et al., 2012; Champagnon, Guillemain, et al., 2012; Söderquist et al., 2017) and other birds (Stojanovic, 2023). For marbled teal, GPS tagging has shown that captive‐bred birds have higher mortality and lower mobility than wild birds after their release, and has provided little evidence that they can breed successfully (Pérez‐García et al., in press). Delaying their release for months after they can fly, in order to avoid the hunting season, can also reduce their ability to adapt to the wild (Green et al., 2005).

Understanding why we did not observe signatures of introgression from the European captive population in the contemporary wild Spanish population could aid future conservation management decisions. Testing the competing hypotheses presented above could be achieved via a multitude of approaches, including a greater level of post‐release monitoring (i.e., via GPS tagging; e.g., Pérez‐García et al., in press) and greater genetic sampling of the wild contemporary population. Delineating populations can have far‐reaching consequences for species conservation, especially for those that already have reduced genetic diversity, we therefore advise greater synergy between current ex situ and in situ conservation management and the continued evaluation of the multifaceted risks associated with any translocation (IUCN/SSC, 2013).

## 5 CONCLUSIONS

Although differentiation of wild populations from Spain and Iraq is limited and coalescent‐based analyses indicate a recent postglacial divergence, the most conservative strategy would be that ex situ conservation programs for marbled teal are exclusively based on the native genetic stock. Unfortunately, genetic management of captive populations of wildfowl do not reach the standards for more charismatic species (e.g., the Iberian Lynx; Kleinman‐Ruiz et al., 2019), and lack of long‐term monitoring of pairings or use of studbooks meant that we obtained limited information about the precise origin of founders and the relationships between the individuals sampled for our study. It is important that the reinforcement program be improved as far as possible to increase population viability (Fundación Biodiversidad, 2022; Pérez‐García et al., in press), implementing long‐term control of pairings, evaluating the evolutionary and genetic risks of captive breeding (i.e., avoiding domestication and loss of genetic diversity; Schulte‐Hostedde & Mastromonaco, 2015), releasing birds at an early age, and monitoring the survival and performance of released birds (e.g., Green et al., 2005). Improved hunting regulations are also vital to reduce mortality after release (Pérez‐García et al., in press). Future studies of wild populations including contemporary samples from other parts of the range—admittedly difficult to obtain—would greatly contribute to obtain a more accurate picture of the genetic composition and connectivity of remnant populations of the marbled teal and allow a better understanding of how their levels of genetic diversity have been impacted by local demographic dynamics.

## CONFLICT OF INTEREST STATEMENT

The authors have no conflicts of interest to declare.

## Supporting information


Data S1.

## Data Availability

All mtDNA sequences have been deposited in GenBank with accession numbers OR798191‐OR798391. Raw Illumina reads have been deposited at the NCBI Sequence Read Archive (SRA) under BioProject PRJNA1037734. Input files for all analyses are available for download on Figshare (https://doi.org/10.6084/m9.figshare.24542464).
