# Supplementary material for: Demographic and conservation genomic assessment of the threatened marbled teal (Marmaronetta angustirostris)
Source: Evol Appl. 2024 May 8;17(5):e13639. doi: 10.1111/eva.13639 (PMC11077658; doi:10.1111/eva.13639)
Supplement: Supplementary file 1 — Data S1. [file EVA-17-e13639-s001.pdf]

Supporting Information for

Demographic and conservation genomic assessment of the threatened marbled teal (*Marmaronetta angustirostris*)

Joaquín Ortego, Violeta Muñoz-Fuentes, Raquel López-Luque, Alex D. Ball, Muhammad Ghazali, Salwan Ali Abed, Mudhafar A. Salim and Andy J. Green

Journal: *Evolutionary Applications*

Contents:

Supplementary tables

METHODS S1 mtDNA amplification and sequencing
METHODS S2 Genomic library preparation
METHODS S3 Genomic data filtering and assembling

Supplementary tables

TABLE S1 Haplotype frequencies for captive and wild populations of marbled teal
TABLE S2 Genetic differentiation between wild populations of marbled teal
TABLE S3 Genetic diversity statistics for captive and wild populations of marbled teal
TABLE S4 Levene's tests for homogeneity of variances in individual genetic diversity
TABLE S5 Mann-Whitney *U* tests for differences in individual genetic diversity

Supplementary figures

FIGURE S1 Alternative demographic models tested using FASTSIMCOAL2
FIGURE S2 Genetic diversity for genotyped populations of marbled teal
FIGURE S3 Log probability of the data and the magnitude of Δ*K* for STRUCTURE analyses
FIGURE S4 Genetic assignment of individuals based on STRUCTURE analyses
FIGURE S5 Co-ancestry heatmap estimated with FINERADSTRUCTURE
FIGURE S6 Pictures of marbled teal with regular and leucistic plumages

References

Supplementary methods

METHODS S1 mtDNA amplification and sequencing

We designed primers L135.MaAn (5´-GTCCCAGTAATACCCATTACCAG-3´) and H614.MaAn (5´-TGGAGGATGCCGCGATTACG-3´) to amplify a 440 bp fragment of the mtDNA control region. For museum specimens, we designed primer H346.MaAn (5´-TAGTGGTTGTCGGGGTATGTCC-3´) which, combined with primer L135.MaAn, amplified a 169 bp fragment containing most polymorphic sites present in the 440 bp sequences obtained for contemporary samples. DNA was amplified using the polymerase chain reaction (PCR) carried out in 25-35 µl reactions containing 1× Buffer II (Applied Biosystems), 2.5 mM $MgCl_2$ (Applied Biosystems), 1 mM dNTPs (0.24 mM each), 0.5 µM of forward and reverse primers, 25-100 ng of genomic DNA and 0.9-1.25 U of AmpliTaq Gold DNA polymerase (Applied Biosystems). PCRs were performed in a T100 PCR Thermal Cycler (Bio-Rad) using the following conditions: one hot-start step of 95 °C for 1 min; 40 cycles of 95 °C for 30 s, 58 °C for 30 s, and 72 °C for 1 min; and a final elongation step of 72 °C for 7 min. When working with extracts from museum specimens, thermal conditions were: one step of 95 °C for 1 min; 42-45 cycles of 95 °C for 30 s, 60 °C for 30 s, and 72 °C for 1 min; and a final elongation step of 72 °C for 7 min. PCR products were run in 2% agarose gels. Both strands of each PCR product were sequenced by Sanger sequencing (Macrogen Inc. Europe). In the case of museum specimens, we sequenced three independent PCR product replicates.

METHODS S2 Genomic library preparation

DNA samples from contemporary samples were processed following the double-digestion restriction-site associated DNA sequencing procedure (ddRAD-seq) adapted from Peterson et al*.,* (2012) and described in Brown et al., (2016) and Dicks et al., (2023) with minor modifications. In brief, DNA quality was assessed via agarose gel electrophoresis on a 1% gel and only samples with a high molecular weight band (assessed against a lambda standard) were selected for the library preparation stage. It should be noted here that DNA quality requirements for this protocol exclude the use of degraded samples (e.g., most historical or non-invasive sample types). DNA was quantified using a Qubit Broad Range dsDNA Assay (Thermo Fisher Scientific) and normalized to 7 ng/µl. We then digested the DNA with the restriction enzymes SbfI and SphI (New England Biolabs, Ipswich, MA, USA) and RAD-specific P1 and P2 paired-end adapters that included a unique pair of 5 or 7 bp barcodes was ligated to each sample. The samples were pooled and then gel electrophoresis extraction was used to select fragments between 400 and 700 bp. Following optimisation of PCR conditions, to identify the minimum number of PCR cycles required to produce sufficient product for sequencing, a bulk amplification of each library (400µl) was conducted. The PCR comprised of 48 µl library template, 12.8 µL combined 10 µM Illumina compatible P1 and P2 adapter specific primers, 200 µl Q5 Hot Start HF 2x Master Mix (New England Biolabs) and 139.2 µL nuclease-free water. Samples were sequenced across two libraries, and positive controls were used to confirm repeatability within and between libraries (Leigh et al., 2018). Each library was sequenced (150 bp, paired-end) on a full lane of an Illumina HiSeq.

METHODS S3 Genomic data filtering and assembling

We used the different programs distributed as part of the STACKS v. 2.62 pipeline (*ustacks*, *cstacks*, *sstacks*, *tsv2bam*, *gstacks* and *populations*) to assemble our sequences into *de novo* loci and call genotypes (Rochette et al., 2019). We assembled reads *de novo* into putative loci with the program *ustacks*. We set the minimum stack depth (*m*) to three and allowed a maximum distance of two nucleotide mismatches (*M*) to group reads into a "stack". We identified single nucleotide polymorphisms (SNPs) at each locus and called genotypes using a multinomial-based likelihood model that accounts for sequencing errors, with the upper bound of the error rate (ε) set to 0.2. We built a catalogue of loci using the *cstacks* program, with loci recognized as homologous across individuals if the number of nucleotide mismatches between consensus sequences (*n*) was ≤2. We used the programs *tsv2bam* and *gstacks* with default parameters to transpose the data to be oriented by locus, integrate paired-end reads to each single-end locus assembled, and call variable sites across individuals. Finally, we exported output files in different formats for subsequent analyses using the program *populations*. Unless otherwise indicated, for all downstream analyses we exported only one random SNP per RAD locus (option *write-random-snp*) and retained loci that were represented in at least 75% of individuals (R = 0.75) and with a minimum minor allele frequency (MAF) ≥ 0.01 (*min_maf* = 0.01).

Supplementary tables

TABLE S1 Haplotype frequencies for captive and wild populations of marbled teal (*Marmaronetta angustirostris*) based on mtDNA control region sequences. Population codes as described in Table 1.

Hap.	cWWTC	cZOOS[a]	cPAJA	cSALE	wGUAD[b]	wGUAD[c]	wVALE[b]	wCAST[c]	wMORO	wTUNI	wALGE	wCAPE	wCHAD	wISRA	wIRAQ	wIRAN
n	10	62	20	23	5	17	32	4	3	1	1	1	1	7	12	2
I	9	62	10	0	0	0	1	0	0	0	0	0	0	0	0	0
II	0	0	10	8	3	0	2	0	0	0	0	0	0	0	0	0
III	0	0	0	12	1	2	9	1	0	1	0	0	0	0	0	0
IV	0	0	0	3	0	9	15	1	2	0	0	1	0	0	0	0
V	0	0	0	0	0	6	5	1	0	0	0	0	0	0	0	0
VI	0	0	0	0	0	0	0	0	0	0	0	0	0	2	2	0
VII	0	0	0	0	0	0	0	0	0	0	0	0	0	0	1	0
VIII	0	0	0	0	1	0	0	1	0	0	0	0	0	0	0	0
IX	0	0	0	0	0	0	0	0	1	0	0	0	1	1	7	1
X	0	0	0	0	0	0	0	0	0	0	0	0	0	1	0	0
XI	0	0	0	0	0	0	0	0	0	0	0	0	0	1	2	0
XII	1	0	0	0	0	0	0	0	0	0	0	0	0	2	0	0
XIII	0	0	0	0	0	0	0	0	0	0	0	0	0	0	0	1
XIV	0	0	0	0	0	0	0	0	0	0	1	0	0	0	0	0

[a]All zoos from continental Europe (cTIER, cKOLN, cOPEL, cGAIA, cCROI, cBRAN, cWALT, and cJERE; see Table 1) present the same haplotype (haplotype I) and were grouped in this table under the code cZOOS; [b]Contemporary samples from Spain (wGUAD: 2005-2020; wVALE: 1991-2020); [c]Historical samples from Spain predating the massive release of captive-bred birds under reinforcement programmes (wGUAD: 1967-1977; wCAST: 1970)

TABLE S2 Genetic differentiation between wild populations ($n \geq 4$) of marbled teal (*Marmaronetta angustirostris*) estimated for (A) the mtDNA dataset (Φ_{ST} statistic) and (B) the ddRAD-seq dataset (F_{ST} statistic). Analyses based on the mtDNA dataset were performed both including all analyzed individuals (above the diagonal) and excluding contemporary samples (1991-2020) from Spain (below the diagonal). Statistically significant values after a false discovery rate (FDR) adjustment (5%) to control for multiple tests are indicated in bold. Population codes as described in Table 1.

(A)	wGUAD	wVALE	wCAST	wISRA	wIRAQ
wGUAD	–	0.000	0.000	**0.288**	**0.162**
wVALE	–	–	0.000	**0.278**	**0.154**
wCAST	0.084	–	–	0.089	0.070
wISRA	**0.375**	–	0.089	–	0.098
wIRAQ	**0.200**	–	0.070	0.098	–

(B)	wGUAD	wVALE	wIRAQ
wGUAD	–		
wVALE	0.032	–	
wIRAQ	**0.055**	**0.043**	–

TABLE S3 Genetic diversity statistics (H_O, H_E, π, and F_{IS}) for captive and wild populations of marbled teal (*Marmaronetta angustirostris*) calculated for the ddRAD-seq dataset considering all positions (polymorphic and nonpolymorphic) and only variant (polymorphic) positions. Statistics are based on a dataset only including unrelated individuals ($\varphi_{ij} \leq 0$; see Figure 3) and considering populations with $n \geq 4$.

					All positions				Variant positions			
**Population**	**Country**[a]	**Status**	**Code**	***n***	H_O	H_E	**π**	F_{IS}	H_O	H_E	**π**	F_{IS}
Wildfowl & Wetlands Trust Centres	UK	Captive	cWWTC	5	0.0004	0.0004	0.0005	0.0002	0.1559	0.1671	0.1904	0.0702
Tierpark Berlin	DE	Captive	cTIER	5	0.0004	0.0004	0.0004	0.0001	0.1553	0.1554	0.1775	0.0461
Parc Animalier de Sainte-Croix	FR	Captive	cCROI	7	0.0003	0.0003	0.0003	0.0002	0.1118	0.1286	0.1401	0.0612
Walter Zoo	CH	Captive	cWALT	4	0.0004	0.0004	0.0004	0.0001	0.1498	0.1447	0.1745	0.0474
Cañada de los Pájaros	ES	Captive	cPAJA	8	0.0004	0.0004	0.0005	0.0001	0.1711	0.1746	0.1883	0.0470
La Granja de El Saler	ES	Captive	cSALE	6	0.0004	0.0004	0.0005	0.0002	0.1721	0.1862	0.2047	0.0726
Guadalquivir Marshes	ES	Wild	wGUAD	4	0.0004	0.0004	0.0005	0.0003	0.1475	0.1718	0.2025	0.1077
Valencia	ES	Wild	wVALE	20	0.0003	0.0005	0.0005	0.0007	0.1232	0.2091	0.2164	0.2899
Iraq	IQ	Wild	wIRAQ	12	0.0005	0.0005	0.0005	0.0002	0.2057	0.2147	0.2257	0.0638

[a]ISO country codes; H_O, observed heterozygosity; H_E expected heterozygosity; π, nucleotide diversity; F_{IS}, Wright's inbreeding coefficient

TABLE S4 Levene's tests for homogeneity of variances in individual genetic diversity, estimated as the proportion of heterozygous loci (i.e., observed heterozygosity, H_O) for the ddRAD-seq dataset. Table shows Levene's statistics (above the diagonal) and *p*-values (below the diagonal) for each pair of populations. Analyses are based on a dataset only including unrelated individuals ($\varphi_{ij} \leq 0$; see Figure 3) and considering populations with $n \geq 4$. For sample sizes and population codes, see Table 1.

	cWWTC	cTIER	cCROI	cWALT	cPAJA	cSALE	wGUAD	wVALE	wIRAQ
cWWTC	–	0.854	0.540	0.135	3.339	0.131	0.027	2.267	8.509
cTIER	0.382	–	0.188	2.225	0.545	0.325	0.664	4.415	2.944
cCROI	0.479	0.674	–	1.811	2.064	0.774	0.529	5.152	7.355
cWALT	0.724	0.179	0.211	–	7.628	0.587	0.009	1.286	18.782
cPAJA	0.095	0.476	0.174	**0.020**	–	1.886	2.194	9.053	1.086
cSALE	0.726	0.583	0.087	0.466	0.195	–	0.196	3.645	5.528
wGUAD	0.875	0.442	0.485	0.929	0.169	0.669	–	1.371	5.135
wVALE	0.146	**0.047**	**0.032**	0.269	**0.006**	0.068	0.254	–	16.133
wIRAQ	**0.011**	0.107	**0.015**	**0.001**	0.311	**0.032**	**0.040**	**0.001**	–

TABLE S5 Mann-Whitney *U* tests for differences in individual genetic diversity, estimated as the proportion of heterozygous loci (i.e., observed heterozygosity, H_O) for the ddRAD-seq dataset. Table shows *U* test statistics (above the diagonal) and *p*-values (below the diagonal) for each pair of populations. Analyses are based on a dataset only including unrelated individuals ($\varphi_{ij} \leq 0$; see Figure 3) and considering populations with $n \geq 4$. For sample sizes and population codes, see Table 1.

	cWWTC	cTIER	cCROI	cWALT	cPAJA	cSALE	wGUAD	wVALE	wIRAQ
cWWTC	–	11	2	7	7	6	8	22	0
cTIER	0.754	–	2	9	0	1	9	24	0
cCROI	**0.012**	**0.012**	–	4	0	0	3	61	0
cWALT	0.462	0.806	0.059	–	3	3	8	27	0
cPAJA	0.057	**0.003**	**0.001**	**0.027**	–	21	5	13	4
cSALE	0.100	**0.011**	**0.003**	0.055	0.699	–	4	11	12
wGUAD	0.624	0.806	**0.038**	1.000	0.062	0.088	–	24	0
wVALE	0.057	0.077	0.619	0.314	**0.001**	**0.003**	0.215	–	3
wIRAQ	**0.002**	**0.002**	**0.001**	**0.004**	**0.001**	**0.025**	**0.001**	**0.001**	–

Supplementary figures

FIGURE S1 Alternative demographic models tested using FASTSIMCOAL2 for wild populations of marbled teal (*Marmaronetta angustirostris*) from Spain (ES, in blue) and Iraq (IQ, in brown). Parameters include mutation-scaled ancestral (ϑ_{ANC}, $\vartheta_{BOT\text{-}ES}$, and $\vartheta_{BOT\text{-}IQ}$) and contemporary ($\vartheta_{ES}$ and ϑ_{IQ}) effective population sizes, migration rates per generation (m_A and m_C), timing of divergence (T_{DIV}), and timing of population size change (T_{BOT}).

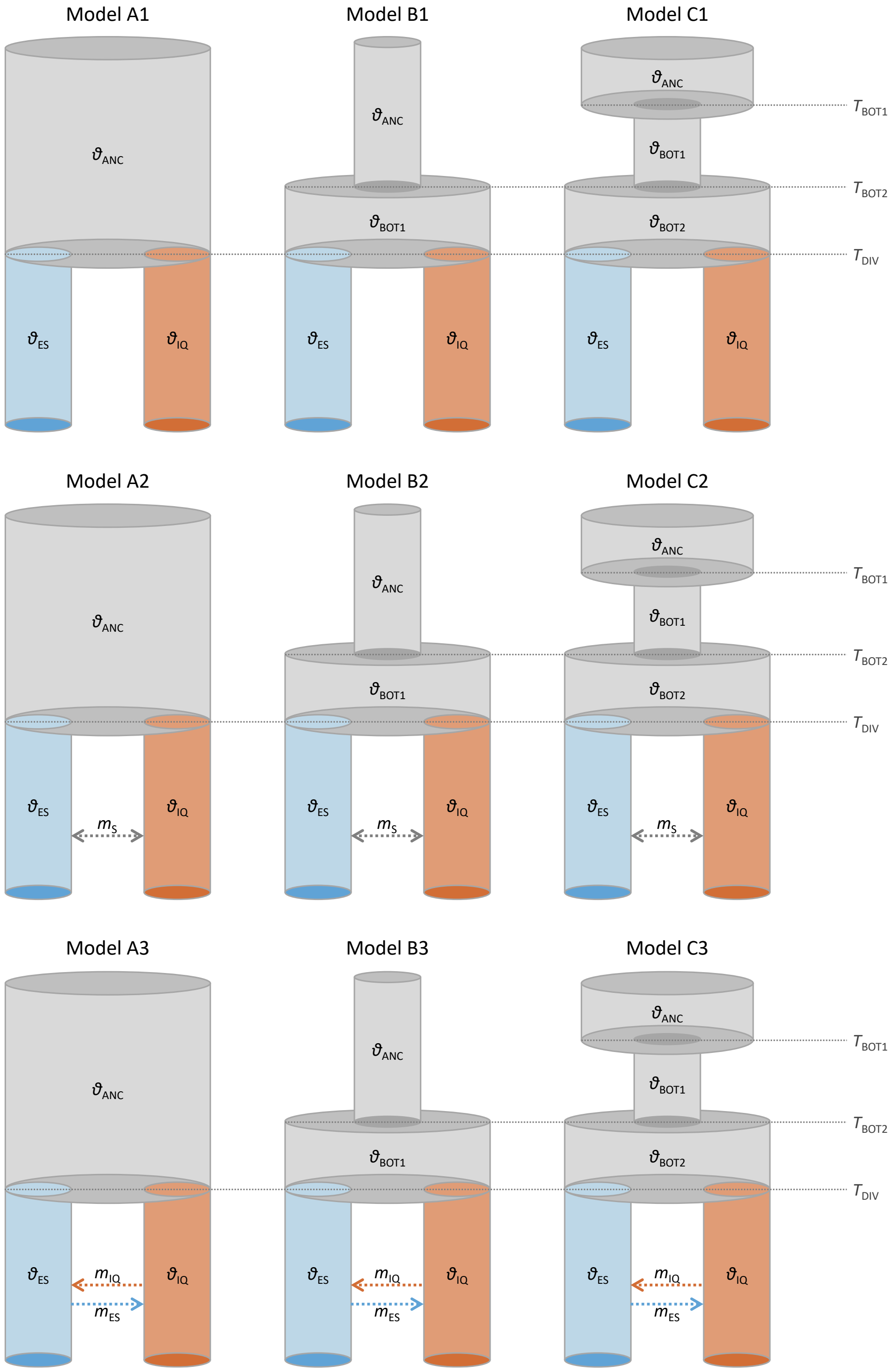

FIGURE S2 Genetic diversity for genotyped populations of marbled teal (*Marmaronetta angustirostris*). Violin plots show estimates of heterozygosity (H_O) for each individual (small coloured dots) and mean and confidence intervals (black dots and vertical bars, respectively) for each population ($n \geq 4$). Plots are based on the whole ddRAD-seq dataset, including both related ($\varphi_{ij} > 0$) and unrelated ($\varphi_{ij} \leq 0$) individuals (see Figure 3). Population codes as described in Table 1.

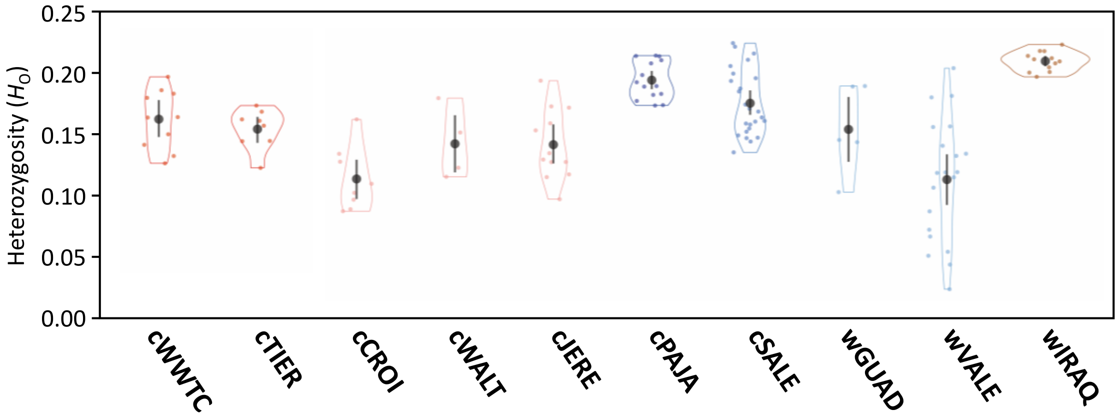

FIGURE S3 Mean (±SD) log probability of the data (LnPr(X|*K*)) over 10 runs of STRUCTURE (left axes, black dots and error bars) for each value of *K* and the magnitude of ΔK (right axes, white triangles). Analyses are based on datasets only including unrelated individuals ($\varphi_{ij} \leq 0$; see Figure 3) and were run for all populations (A) and separately for wild populations (B).

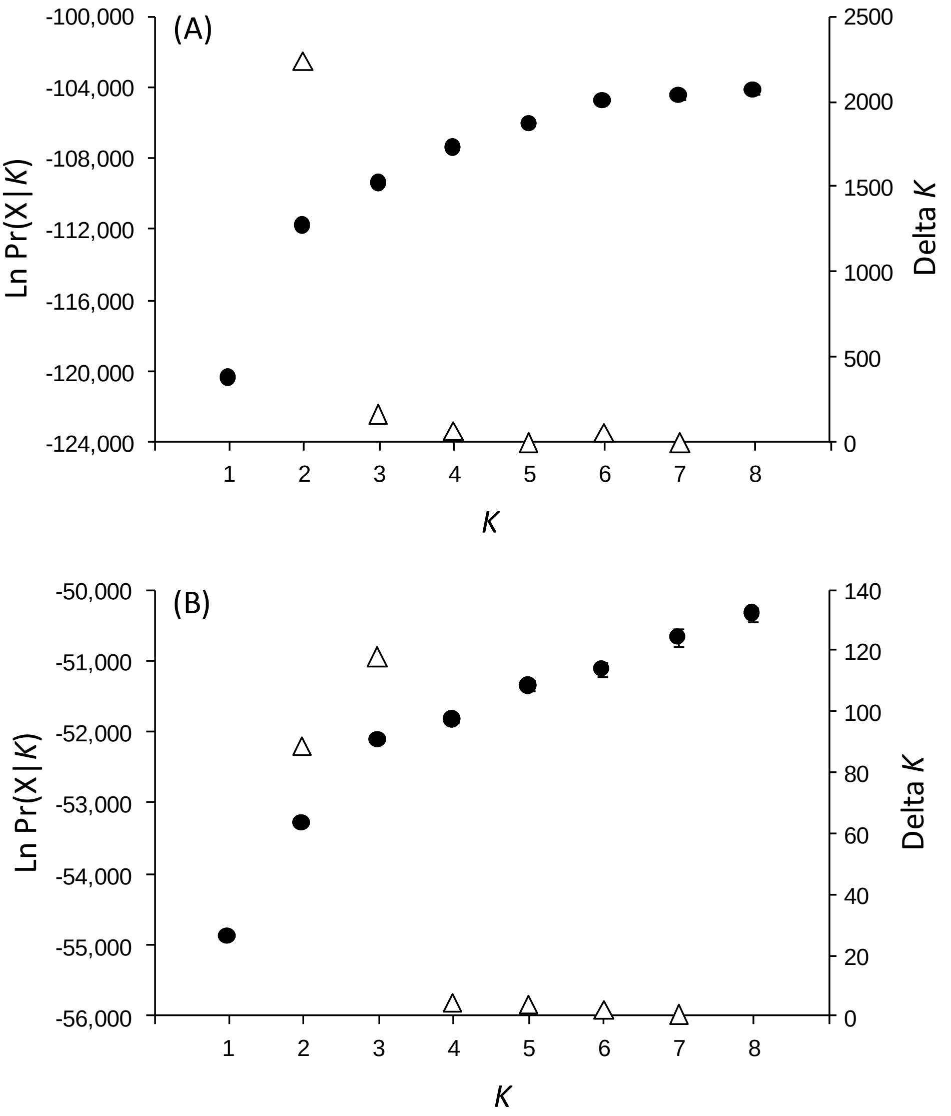

FIGURE S4 Results of genetic assignments based on the program STRUCTURE for captive and wild populations of marbled teal (*Marmaronetta angustirostris*). Analyses are based on datasets only including unrelated individuals ($\varphi_{ij} \leq 0$; see Figure 3) and were run for all populations (A) and separately for wild populations (B). Each individual is represented by a vertical bar partitioned into *K* coloured segments showing the individual's probability of belonging to the cluster with that colour. Vertical black lines separate individuals from different populations. Population codes as described in Table 1.

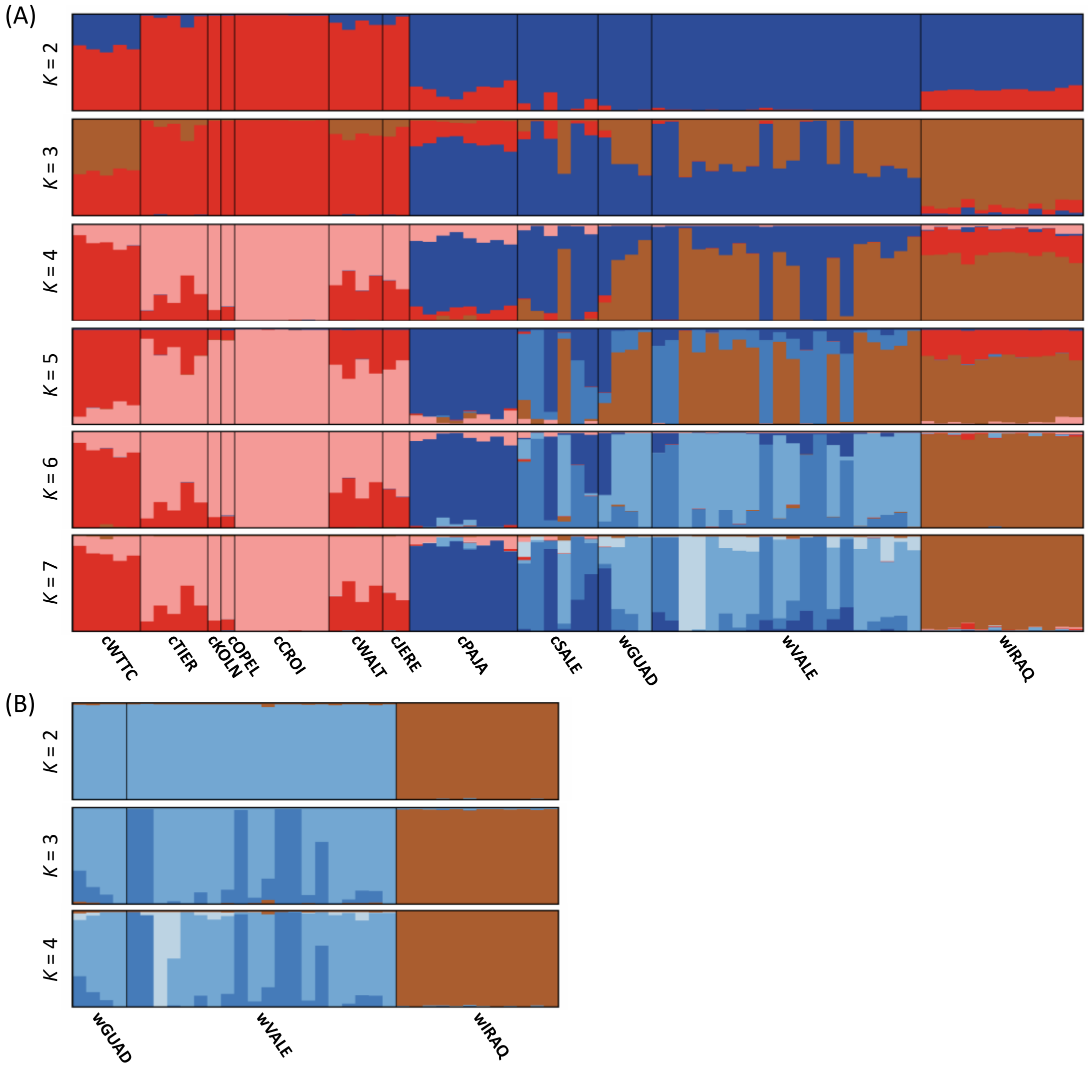

FIGURE S5 Simple co-ancestry heatmap for captive and wild populations of marbled teal (*Marmaronetta angustirostris*) estimated with FINERADSTRUCTURE. Pairwise coefficients of co-ancestry are colour coded from low (yellow) to high (black). Colour bars on the bottom indicate the three main population groups: European zoo populations (cWWTC, cTIER, cKOLN, cOPEL, cCROI, cWALT, and cJERE), wild (wGUAD, wVALE) and captive (cPAJA, cSALE) populations from Spain, and wild populations from Iraq (wIRAQ). Analyses are based on a dataset only including unrelated individuals ($\varphi_{ij} \leq 0$; see Figure 3). Population codes as described in Table 1.

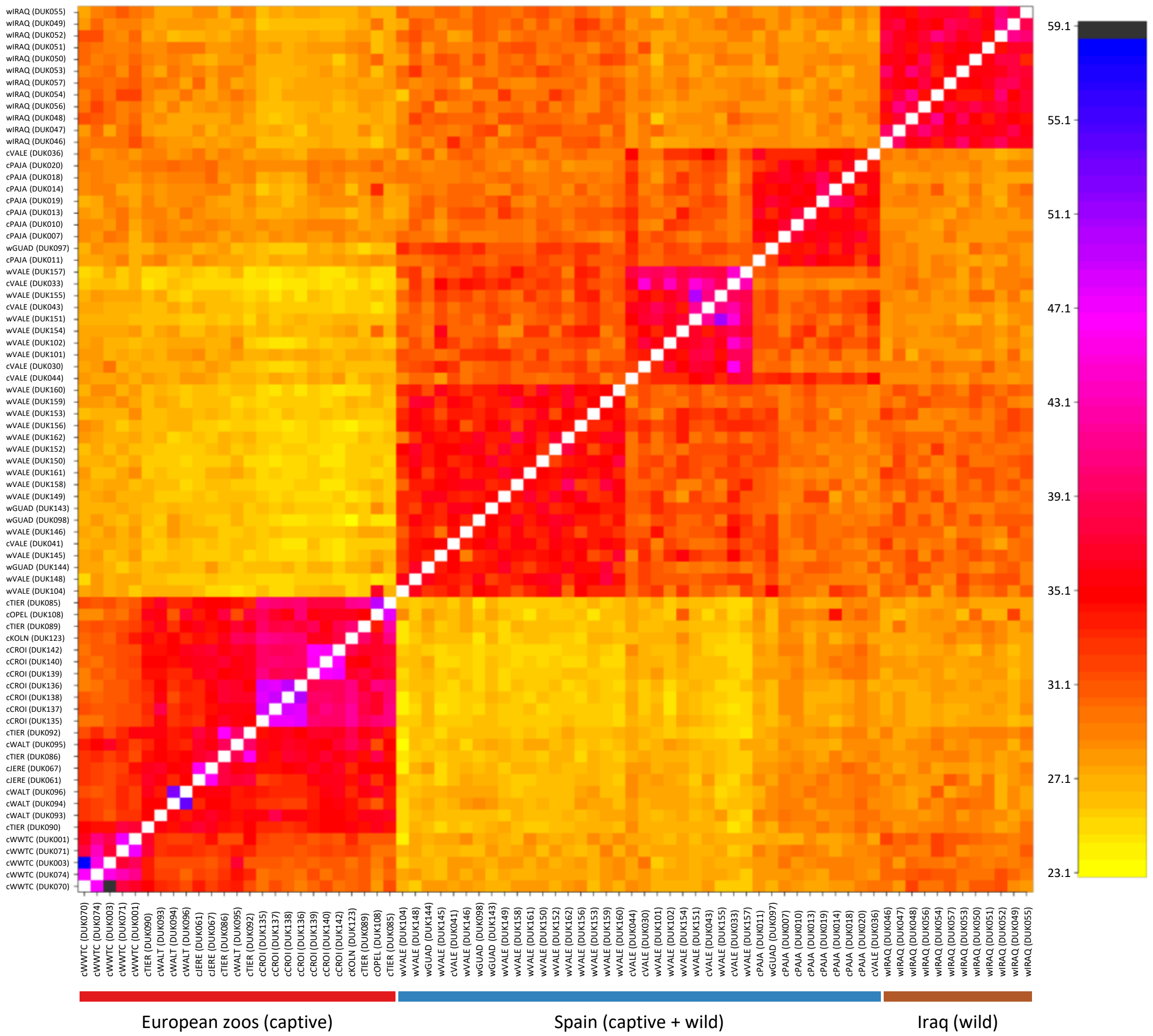

FIGURE S6 Pictures of marbled teal (*Marmaronetta angustirostris*) with regular and leucistic plumages. Panel (A) shows two birds from El Hondo wetland (Valencia, Spain), one with leucistic plumage (top) and another with regular plumage (bottom). Panel (B) shows one bird with leucistic plumage from El Clot de Galvany wetland (Valencia, Spain). Pictures by Juan Manuel Pérez García, taken in 2020.

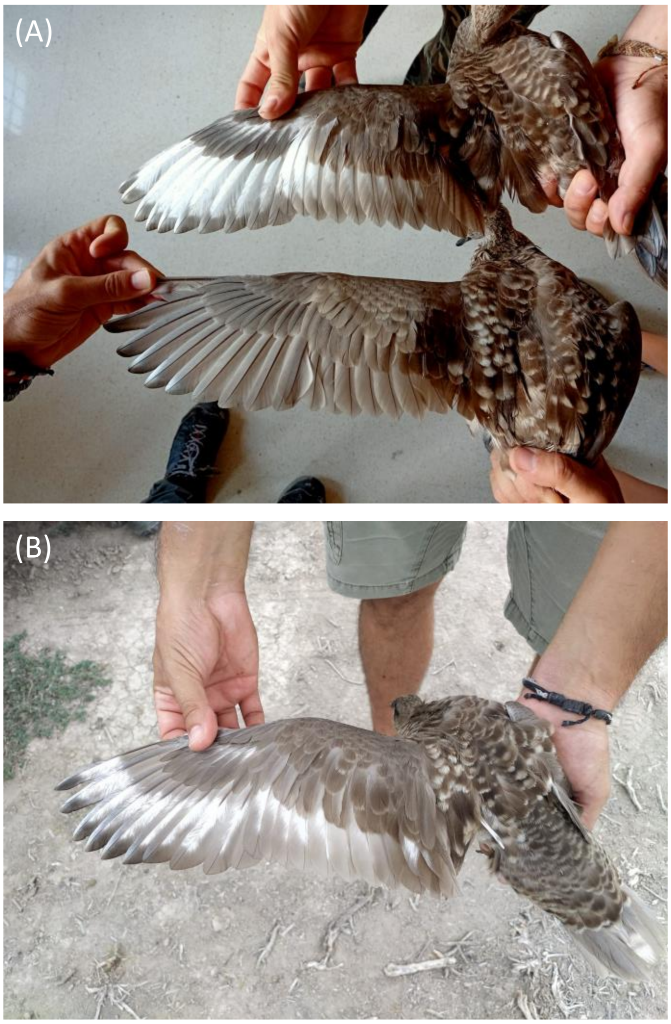

References

Brown, J. K., Taggart, J. B., Bekaert, M., Wehner, S., Palaiokostas, C., Setiawan, A. N., . . . Penman, D. J. (2016). Mapping the sex determination locus in the hapuku (*Polyprion oxygeneios*) using ddRAD sequencing. *BMC Genomics, 17*, 448. doi:10.1186/s12864-016-2773-4

Dicks, K. L., Ball, A. D., Banfield, L., Barrios, V., Boufaroua, M., Chetoui, A., . . . Gilbert, T. (2023). Genetic diversity in global populations of the critically endangered addax (*Addax nasomaculatus*) and its implications for conservation. *Evolutionary Applications, 16*(1), 111-125. doi:10.1111/eva.13515

Leigh, D. M., Lischer, H. E. L., Grossen, C., & Keller, L. F. (2018). Batch effects in a multiyear sequencing study: False biological trends due to changes in read lengths. *Molecular Ecology Resources, 18*(4), 778-788. doi:10.1111/1755-0998.12779

Peterson, B. K., Weber, J. N., Kay, E. H., Fisher, H. S., & Hoekstra, H. E. (2012). Double digest RADseq: An inexpensive method for *de novo* SNP discovery and genotyping in model and non-model species. *PLoS One, 7*(5), e37135. doi:10.1371/journal.pone.0037135

Rochette, N. C., Rivera-Colón, A. G., & Catchen, J. M. (2019). STACKS 2: Analytical methods for paired-end sequencing improve RADseq-based population genomics. *Molecular Ecology, 28*(21), 4737-4754. doi:10.1111/mec.15253
